# Achieving Ultra-Broad Microwave Absorption Bandwidth Around Millimeter-Wave Atmospheric Window Through an Intentional Manipulation on Multi-Magnetic Resonance Behavior

**DOI:** 10.1007/s40820-024-01395-4

**Published:** 2024-04-22

**Authors:** Chuyang Liu, Lu Xu, Xueyu Xiang, Yujing Zhang, Li Zhou, Bo Ouyang, Fan Wu, Dong-Hyun Kim, Guangbin Ji

**Affiliations:** 1 School of Materials Science and Technology, Nanjing University of Aeronautics and Astronautics, Nanjing, 210016 Jiangsu People’s Republic of China; 2 School of Materials Science and Engineering, Nanjing University of Science and Technology, Nanjing, 210094 Jiangsu People’s Republic of China; 3 School of Physics, Nanjing University of Science and Technology, Nanjing, 210094 Jiangsu People’s Republic of China; 4 School of Mechanical Engineering, Nanjing University of Science and Technology, Nanjing, 210094 Jiangsu People’s Republic of China; 5 School of Physics, Chungbuk National University, Cheongju, 28644 South Korea; 6 Department of Chemistry, School of Science, Tianjin University, Tianjin 300072, People’s Republic of China

**Keywords:** Microwave absorption, Ultra-broad bandwidth, M-type barium ferrite, Magnetocrystalline anisotropy field, Multi-magnetic resonance

## Abstract

The utilization of electromagnetic waves is rapidly advancing into the millimeter-wave frequency range, posing increasingly severe challenges in terms of electromagnetic pollution prevention and radar stealth. However, existing millimeter-wave absorbers are still inadequate in addressing these issues due to their monotonous magnetic resonance pattern. In this work, rare-earth La^3+^ and non-magnetic Zr^4+^ ions are simultaneously incorporated into M-type barium ferrite (BaM) to intentionally manipulate the multi-magnetic resonance behavior. By leveraging the contrary impact of La^3+^ and Zr^4+^ ions on magnetocrystalline anisotropy field, the restrictive relationship between intensity and frequency of the multi-magnetic resonance is successfully eliminated. The magnetic resonance peak-differentiating and imitating results confirm that significant multi-magnetic resonance phenomenon emerges around 35 GHz due to the reinforced exchange coupling effect between Fe^3+^ and Fe^2+^ ions. Additionally, Mössbauer spectra analysis, first-principle calculations, and least square fitting collectively identify that additional La^3+^ doping leads to a profound rearrangement of Zr^4+^ occupation and thus makes the portion of polarization/conduction loss increase gradually. As a consequence, the La^3+^–Zr^4+^ co-doped BaM achieves an ultra-broad bandwidth of 12.5 + GHz covering from 27.5 to 40 + GHz, which holds remarkable potential for millimeter-wave absorbers around the atmospheric window of 35 GHz.

**Supplementary Information:**

The online version contains supplementary material available at 10.1007/s40820-024-01395-4.

## Highlights

- The frequency and intensity of multi-magnetic resonance are freely regulated by co-doping La^3+^ and Zr^4+^ ions.
- Zr^4+^ occupation is elaborately modified for promoting the portion of polarization/conduction loss to increase profoundly.
- The optimized electromagnetic characteristics lead to an ultra-wide bandwidth of 12.5+ GHz around millimeter-wave atmospheric window.

**Supplementary Information:**

The online version contains supplementary material available at 10.1007/s40820-024-01395-4.

## Graphical abstract

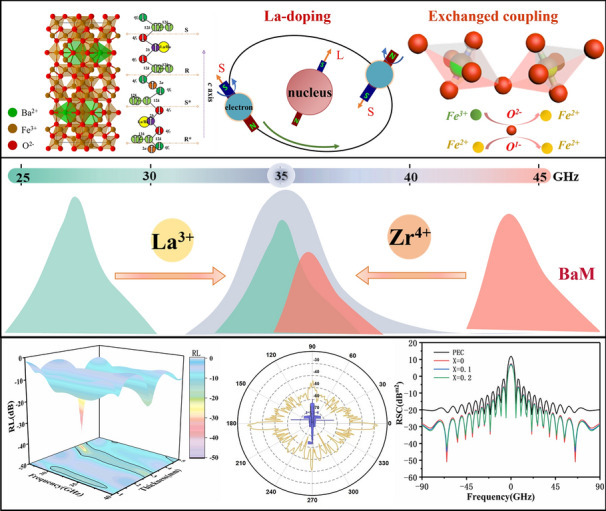

## Introduction

The rapid development of 5G technology has led to a new booming situation in various high-tech industries. This is primarily due to the incorporation of the millimeter-wave range of 25–52.6 GHz in FR2, which offers advantages such as high energy and wide bandwidth for faster network speeds [1–4]. Meanwhile, thanks to its superior attributes including high resolution and all-weather capabilities, the millimeter-wave around atmospheric window of 35 GHz has emerged as an optimal choice for next-generation radar detection [5]. However, this progress has also brought about increasingly severe electromagnetic pollution and national security concerns. Consequently, there is an urgent need for research on millimeter-wave absorbing materials, specifically designed for the atmospheric window of 35 GHz.

As an exceptional millimeter absorber, the primary characteristic lies in its possession of appropriate magnetic or dielectric losses within the millimeter band. M-type barium ferrite BaFe_12_O_19_ (BaM) is a magic material known for numerous advantages such as easy fabrication and environmental-friendly. Especially, its high magnetocrystalline anisotropy field (*H*_a_) leads to the generation of natural resonance around 45 GHz, approaching millimeter-wave atmospheric window of 35 GHz. These distinctive qualities make it a highly sought-after choice for extensive applications in millimeter-wave absorption [6–8]. However, the solitary natural resonance of original barium ferrite usually results in a limited absorption bandwidth, which cannot meet the ever-increasing requirements for millimeter-wave absorption applications. Previous studies have shown that part of Fe^3+^ ions would transform into Fe^2+^ ions in BaM by replacing Fe^3+^ ions with high-valence Zr^4+^, Ti^4+^, Nb^5+^ ions for charge balance. The exchange coupling effect of Fe^3+^, Fe^2+^ and O^-^ ions generates new Lande *g*-factors in M-type barium ferrite, which is different with that of intrinsic Fe^3+^ ions. The coexistence of multiple *g*-factors leads to the emergence of multiple resonance loss peaks for a significant expansion in magnetic loss range. Consequently, the broad absorption bandwidth can be attained due to the concurrently improved impedance matching and attenuation capacity [5, 9, 10]. Nevertheless, the substitution of non-magnetic high-valence ions for Fe^3+^ ions will lead to a reduction of *H*_a_, accompanying with the natural resonance frequency moving toward the lower frequency range monotonically. Ultimately, it results in an unalterable relationship between natural resonance frequency and exchange coupling strength of Fe^3+^, Fe^2+^, and O^-^ ions, rather than allowing for independent and unrestricted adjustments. As a result, the exchange coupling strength is constrained at specific 35 GHz atmospheric window by solely non-magnetic high-valence ions doping. The lack of strength modification thus hinders the potential enhancement of millimeter-wave absorption performance.

In light of the aforementioned issue, this work aims to develop a high-efficiency millimeter-wave absorber that targets the atmospheric window of 35 GHz via overcoming the restrictive relationship between natural resonance frequency and exchange coupling strength. To achieve this goal, non-magnetic Zr^4+^ ions and rare-earth La^3+^ ions are simultaneously incorporated into BaM. By doping high-valence ions at dual positions, Fe^2+^ concentration can be further boosted to enhance the exchange coupling strength among Fe^3+^, Fe^2+^, and O^-^. Moreover, due to its unique electronic layer structure, rare-earth La^3+^ exhibits characteristics such as high anisotropy field and atomic magnetic moment [11]. In contrast to non-magnetic Zr^4+^ ions, the presence of rare-earth La^3+^ ions has an increasing impact on the natural resonance frequency. Therefore, based on the opposite effect of La^3+^ and Zr^4+^ ions on *H*_a_, the natural resonance peak of BaM can be modulated to the atmospheric window of 35 GHz by precisely regulating the doping ratio of La^3+^ and Zr^4+^ ions. It is thus expected to make an independent regulation on frequency and intensity of multi-magnetic resonance by co-doping non-magnetic Zr^4+^ ions and rare-earth La^3+^ ions, reinforcing the absorbing performance in a given target frequency band theoretically.

Previous studies have shown that doping BaM with Zr^4+^ ions in a chemical composition of BaZr_0.3_Fe_11.7_O_19_ enables effective regulation of absorption range slightly below the millimeter-wave atmospheric window of 35 GHz [12]. Herein, we present an elaborately designed La^3+^–Zr^4+^ ions co-doped barium ferrite (LBZFO) with a consistent Zr^4+^ content of La_*x*_Ba_1-*x*_Zr_0.3_Fe_11.7_O_19_ (*x* = 0–0.2), using a facile sol–gel route which has the advantages of producing homogeneous product compositions and facilitating the synthesis of intricate inorganic compounds. The results demonstrate significant multi-magnetic resonance phenomenon around 35 GHz through intentional manipulation strategy employed in this work. Meanwhile, the portion of polarization/conduction loss elevates gradually with an increment of La^3+^ content for improved impedance matching purposes. Eventually, the LBZFO sample with *x* = 0.1 exhibits extraordinary absorption performance within the millimeter-wave atmospheric window of 35 GHz with an ultra-broad bandwidth of 12.5 + GHz covering from 27.5 to 40 + GHz. Overall, this work provides valuable guidance to develop high-efficiency millimeter-wave absorber.

## Experimental Section

### Materials

Lanthanum nitrate, barium nitrate, zirconium nitrate, ferric nitrate, ammonia water, citric acid and anhydrous ethanol were procured from Shanghai Aladdin Industrial Corporation. All reagents were employed as received without any further purification.

### Preparation

Firstly, lanthanum nitrate, barium nitrate, zirconium nitrate and ferric nitrate were weighed according to the stoichiometric proportions and dissolved in deionized water under constant magnetic stirring for 0.5 h to form a clear solution. Subsequently, ammonia water was added to adjust the pH value to 7, and citric acid was used for sufficient complexation reaction to get a stable and transparent sol. Afterward, the obtained sol was heated and stirred at 80 °C for 3 h to get a wet gel, which was then transformed into a dry gel with a fluffy state by being placed in an oven at 120 °C for 3 days. Next, the dry gel was calcined at 450 °C on a heating platform, resulting in red-brown powders in the forms of intermediate phases La^3+^-doped BaCO_3_ and Zr^4+^ doped Fe_2_O_3_. The attained precursors were sintered at 1300 °C for 3 h with a heating rate of 5 °C min^-1^ to finally achieve the desired La^3+^–Zr^4+^ double-position co-doped barium ferrite powders.

### Characterizations

The phase structures and microstructures of the La^3+^–Zr^4+^ co-doped barium ferrite powders were identified by X-ray diffraction (XRD, PANalytical B V Empyrean 200,895, Cu Ka radiation) and scanning electron microscopy (SEM, Hitachi SU-70 FESEM), separately. Hysteresis loops were measured by a magnetic property measurement system (MPMS-XL-5). The element states of Fe and O were recognized by using X-ray photoelectron spectroscopy (XPS, PHI 5000 Versa probe). The Raman spectrometer (Renishaw In Via 2000) and Mössbauer Spectrometer (MFD-500AV-03) were employed to study the occupation sites of Fe ions in barium ferrite. The electromagnetic parameters in the frequency range of 26.5–40 GHz (R band) were carried out by an vector network analyzer (Ceyear 3672C).

### Calculations and Simulations

All density functional theory (DFT) calculations were conducted using the Ab-initio Software Package (VASP) program with Perdew–Burke–Ernzerhof (PBE) exchange–correlation functional with spin polarization. On the basis of the experimental results, different types of doping were applied as calculated models using single cells of ferrite. A Monkhorst–Pack grid size of 5 × 5 × 1 was employed for surface calculations. The cut-off energy for plane wave expansions was set at 520 eV. Convergence tolerance optimization was performed for energy and force with values set at 1.0 × 10^-6^ eV and 0.01 eV Å^-1^ respectively.

To analyze millimeter-wave propagation characteristics within the sample, near-field simulations were carried out using CST Studio Suite 2020 software package. The electric boundary was set as open boundary condition, and the excitation frequency was carefully chosen at 35 GHz. Considering practical application requirements, a simplified Predator II UAV (unmanned aerial vehicle) model was used for far-field radar cross-section (RCS) simulations. Detailed calculations were conducted to determine the interaction of incident millimeter-waves with the absorbing coating on the UAV model at different angles and motion states.

## Results and Discussion

### Preparation and Characterization of the LBZFO

#### Phase, Morphology and Element State of the LBZFO

**Fig. 1:**
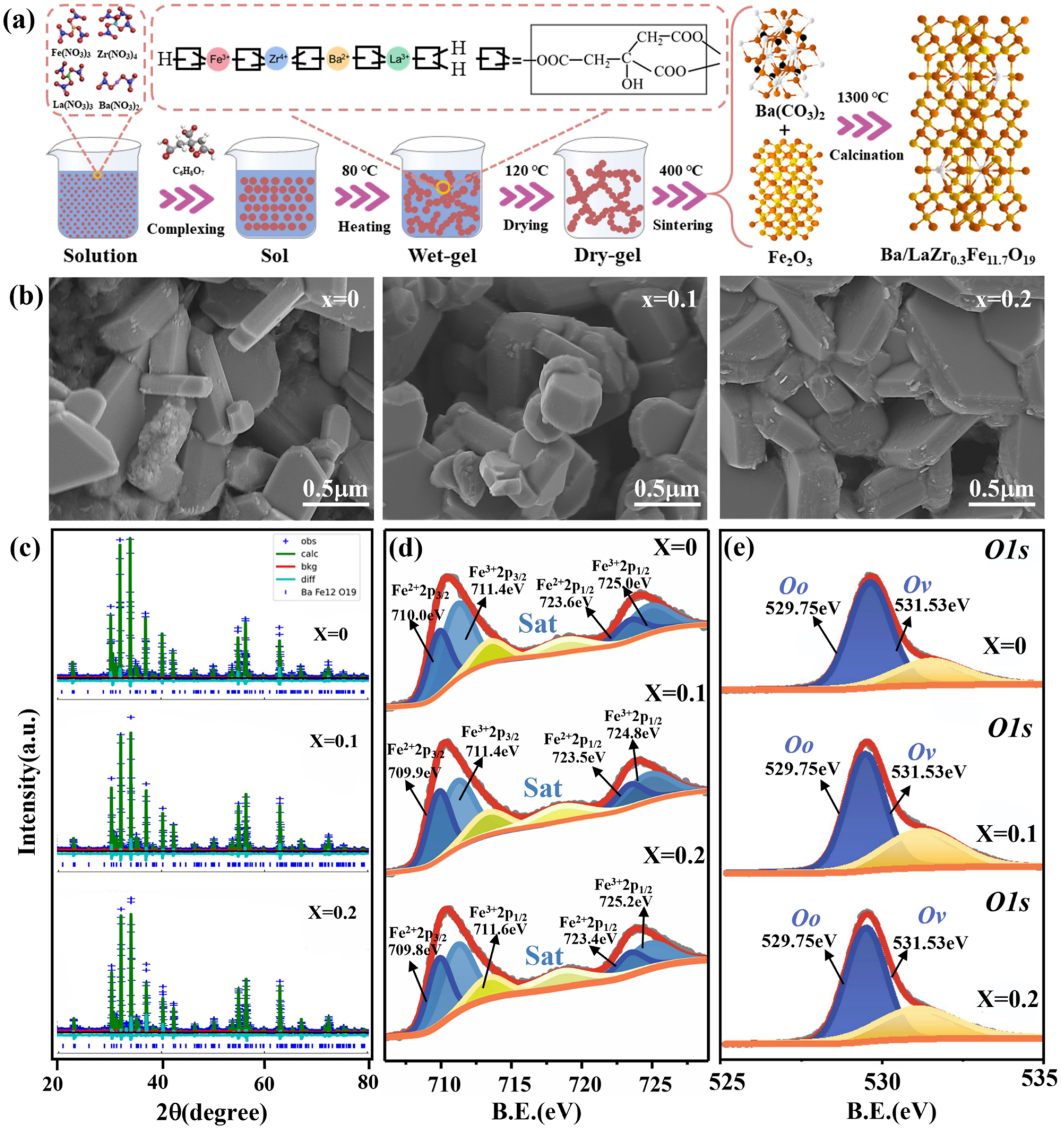
**a** Preparation flow chart, **b** SEM images, **c** rietveld refinement of XRD and **d** XPS spectra for Fe *2p* and O 1*s* of the La_*x*_Ba_1-*x*_Zr_0.3_Fe_11.7_O_19_ samples with *x* = 0, 0.1, 0.2

The LBZFO samples with chemical formula of La_*x*_Ba_1-*x*_Zr_0.3_Fe_11.7_O_19_ (*x* = 0–0.2) were synthesized using a sol–gel process, and the detailed flow chart of the preparation is shown in Fig. 1a. Additionally, Fig. 1b presents SEM images of the as-prepared LBZFO samples with varying proportions of La^3+^ ions. As can be observed, each sample exhibits a characteristic hexagonal plate-like microstructure, consistent with the morphology of original BaM (Fig. S1) [9, 13]. The irregular accumulation of hexagonal plate-like grains inevitably leads to numerous voids formation, which could potentially enhance multi-scattering effects for microwaves in the LBZFO samples. Besides, it is noteworthy that the grain size of all LBZFO samples remains similar despite extra-doping La^3+^ ions into BaM lattice structure, thus suggesting minimal impact on its morphology by these additional dopants.

To precisely determine the phase composition, Rietveld refinement analysis was performed on XRD patterns by GSAS-II software as depicted in Fig. 1c. Clearly, successful attainment of M-type barium ferrite phase is confirmed without any obvious impurity phases related to La or Zr elements detected across all samples examined here. The implication here suggests that it is highly probable for foreign La^3+^ or Zr^4+^ ions integrating into the lattice structure of BaM phase. Virtually, the respective ionic radii values of La^3+^, Ba^2+^, Fe^3+^, and Zr^4+^ are 1.06, 1.34, 0.64, and 0.72 Å [10, 12, 14–16]. Based on the principle of radius similarity, it is initially speculated that La^3+^ might substitute for Ba^2+^; while, Zr^4+^ replace Fe^3+^ in BaM. Furthermore, the Rietveld refinement results pertaining to lattice constants and cell volumes of the LBZFO samples are listed in Table 1. Compared with those of original BaM summarized in Fig. S2, it is seen that the lattice constants “a”, “c” and cell volume increase from 5.893 Å, 23.207 Å and 697.941 Å^3^ of the original BaM to 5.904 Å, 23.313 Å and 703.862 Å^3^ by initially Zr^4+^ ions doping to BaZr_0.3_Fe_11.7_O_19_. While they subsequently show a continuous reduction to 5.898 Å, 23.265 Å and 700.912 Å^3^ with an increment of La^3+^ doping content to *x* = 0.2 of La_0.2_Ba_0.8_Zr_0.3_Fe_11.7_O_19_. The initially increased lattice constants and cell volume provide a powerful evidence for the fact that Zr^4+^ ions (0.72 Å) primarily take the place of smaller Fe^3+^ (0.64 Å) ions, whilst the following decrease in lattice constants and cell volume confirm that La^3+^ ions (1.06 Å) tend to occupy the position of larger Ba^2+^ ions (1.34 Å) in the BaM lattice.

**Table 1:** Lattice constants a, c and cell volumes of La_*x*_Ba_1-*x*_Zr_0.3_Fe_11.7_O_19_ (*x* = 0–0.2)

*x*	a (Å)	c (Å)	V (Å)^3^
0	5.904	23.313	703.862
0.1	5.902	23.299	703.036
0.2	5.898	23.265	700.912

The XPS spectra of Fe 2*p* and O 1*s* for the LBZFO samples and the pure BaM were thoroughly measured to determine the changes in concentrations of Fe^2+^ and oxygen vacancy induced by Zr^4+^ and La^3+^ ions doping. The Fe 2*p*, O 1*s* spectra of all specimens exhibit characteristic peaks of Fe^2+^ 2*p*_3/2_, Fe^3+^ 2*p*_3/2_, Fe^2+^ 2*p*_1/2_, and Fe^3+^ 2*p*_1/2_ at ~ 709.9, ~ 711.5, ~ 723.5, and ~ 725.0 eV, and lattice oxygen, oxygen vacancy at ~ 530.0 and ~ 531.5 eV respectively (Figs. 1d, e and S3a, b). These resolved peaks positions are consistent well with previous reports on the binding energy of Fe^3+^, Fe^2+^, lattice oxygen and oxygen vacancy in XPS [17, 18]. Meanwhile, the proportions of Fe^2+^ ions and oxygen vacancy in the barium ferrite were calculated according to the corresponding peak areas (Table 2 and Fig. S3). Apparently, both the contents of Fe^2+^ ions and oxygen vacancy boost with increasing Zr^4+^ or La^3+^ ions doping. This observation can be attributed to a charge balance mechanism, where the lattice ions being replaced by higher-priced foreign ions would cause a part of Fe^3+^ ions to change into Fe^2+^ ions in the ferrites [19]. The results hence further confirm the aforesaid fact that Zr^4+^/La^3+^ ions are substituting for Fe^3+^/Ba^2+^ in BaM respectively as the following expressed defect reactions:

1 $${\text{ZrO}}_{2} + {\text{Fe}}_{{{\text{Fe}}}} \mathop{\longrightarrow}\limits^{{{\text{BaFe}}_{12} {\text{O}}_{19} }}Zr_{{{\text{Fe}}}}^{ \cdot } + {\text{Fe}}_{{{\text{Fe}}}}^{\prime } + 2{\text{O}}_{{\text{O}}}$$

2 $${\text{La}}_{2} {\text{O}}_{3} + 2{\text{Fe}}_{{{\text{Fe}}}} \mathop{\longrightarrow}\limits^{{{\text{BaFe}}_{12} {\text{O}}_{19} }}2{\text{La}}_{{{\text{Ba}}}}^{ \cdot } + 2Fe_{{{\text{Fe}}}}^{\prime } + 3{\text{O}}_{{\text{O}}}$$

**Table 2:** Fe^2+^ and oxygen vacancy contents of La_*x*_Ba_1-*x*_Zr_0.3_Fe_11.7_O_19_(*x* = 0–0.2)

*x*	Y	C(Fe^2+^) (%)	C(OV) (%)
0	0.3	37.44	29.30
0.1	0.3	38.89	36.54
0.2	0.3	41.36	37.32

Meanwhile, the oxygen vacancy also could be induced in the ferrites to maintain the stability of the crystal lattice after foreign ions doping [20], which could benefit the generation of extensive electric dipoles in the LBZFO samples.

#### ***Occupation Analysis of Zr***^***4***+^***for Fe***^***3***+^***in the LBZFO Structure***

**Fig. 2:**
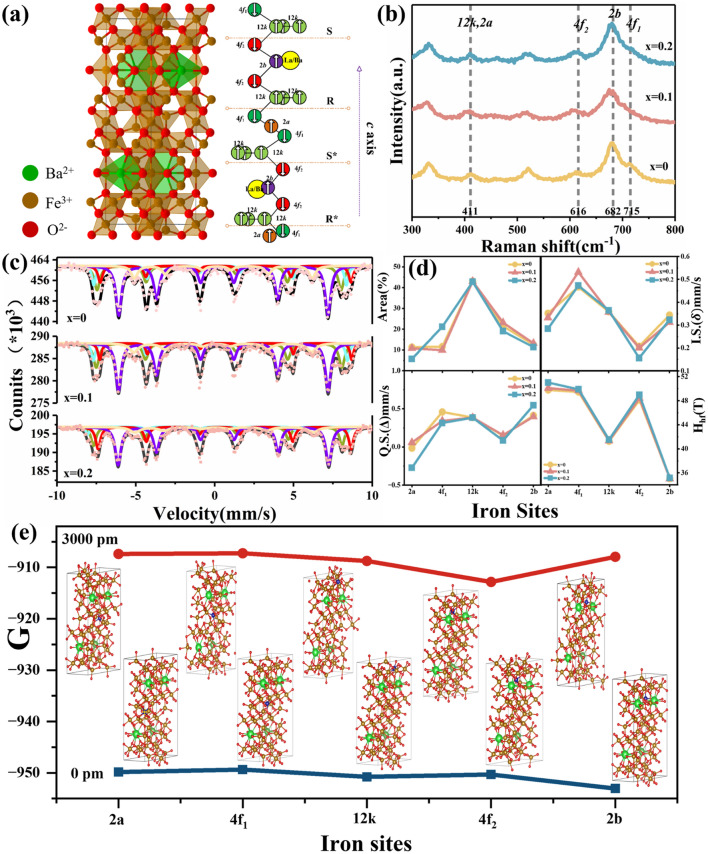
**a** Lattice structure diagram of BaM, **b** Raman patterns, **c** Mössbauer spectra, **d** parameters of occupation area, I.S., Q.S., *H*_*hf*_ deduced from Mössbauer spectra of the La_*x*_Ba_1-*x*_Zr_0.3_Fe_11.7_O_19_ samples with *x* = 0, 0.1, 0.2 and **e** theoretical calculations of enthalpy for the La^3+^ and Zr^4+^ co-doped barium ferrite with Zr^4+^ substituting for Fe^3+^ at various sites based on first principles

In fact, as shown in Fig. 2a, the O^2-^ ions adopt diverse oxygen polyhedral structures (octahedral, tetrahedral, and triangular bipyramids) within the lattice structure of BaM. As a result, Fe^3+^ ions occupy five distinct sublattice positions (12k, 2a, 2b, 4f_1_ and 4f_2_) due to variations in their surrounding oxygen environments. The contribution of Fe^3+^ ions at these five positions exhibits pronounced disparities in magnetic properties, including *H*_a_, magnetic saturation strength (*M*_s_) and etc. [21, 22]. Therefore, for a comprehensive understanding of the regulation mechanism underlying millimeter-wave absorption through Zr^4+^–La^3+^ ions doping, it is crucial to precisely identify which Fe^3+^ sites are substituted by Zr^4+^ ions and how this substitution varies with changes in doping content. By analyzing the Raman spectra shown in Fig. 2b, it can be affirmed that La^3+^ ions doping significantly affects the position distribution of Zr^4+^ substituting for Fe^3+^. To our knowledge, a single unit cell of BaM contains a total of 64 atoms to generate 189 optical modes, and there exist a total of 42 Raman active optical modes (11A_1g_ + 14E_1g_ + 17E_2g_) based on the D_6h_ factor group symmetry [23, 24]. Among them, the characteristic peak at 682 cm^-1^ belongs to the triangular-bipyramidal 2b site of Fe^3+^ ions. The scattering peak near 715 cm^-1^ is ascribed to the tetrahedral 4f_1_ site; while, the peaks at 411 cm^-1^ and 616 cm^-1^ are attributed to the octahedral 12k/2a and 4f_2_ sites induced by the A_1g_ mode. Through a comparison with pure BaM in Fig. S4, the intensity of the scattering peaks at 715 and 411 cm^-1^ changes noticeably after doping Zr^4+^ to composition of BaZr_0.3_Fe_11.7_O_19_, suggesting that the initial addition of Zr^4+^ ions primarily occupy the Fe^3+^ ions at 4f_1_ and (12k or 2a) sites. While a slight change for the peak at 616 cm^-1^ emerge then after further doping La^3+^ ions. It is inferred that the subsequent La^3+^ ions doping has a non-negligible impact on Fe^3+^ ions at 4f_2_ position.

To further investigate the specific effect of La^3+^ ions doping on the occupation position distribution of Zr^4+^ for Fe^3+^, Mössbauer spectra were obtained for both the original BaM and LBZFO samples. The well-fitted results, consisting of five magnetic sextets, are presented in Figs. S5 and 2c, respectively. Considering the structural position multiplicity and the resonance absorption coefficient of Fe at each position (12k, 4f_1_, 4f_2_, 2a, 2b), the theoretical ratio of these five positions is identified to be 50:17:17:8:8 [25, 26]. According to Fig. S6a, it can be seen that the proportion distribution of Fe^3+^ sites in pure BaM sample closely matches with the theoretical value. However, the proportion of the five positions varies significantly after doping behavior as exhibited in Fig. 2d. Specifically, for the component of BaZr_0.3_Fe_11.7_O_19_, it is confirmed that Zr^4+^ ions preferentially occupy positions at 12k and 4f_1_ initially due to their location far from the barium layer which makes them less bound and hence more easily replaced by doping ions [27]. With an increase content of La^3+^ ions up to *x* = 0.2, there is a substantial increase in Fe occupancy at site 4f_1_ even exceeding that observed for pure BaM; while, Fe at 2a and 4f_2_ sites reveal an apparent decreasing trend concurrently. It indicates that the additional doping of La^3+^ ions leads to a marked rearrangement of Zr^4+^ ion occupation from 4f_1_ site toward 2a and 4f_2_ sites. Besides, the Mössbauer spectra also provide the information on hyfine interaction of Fe, including electric monopole interaction, electric quadrupole interaction and magnetic dipole interaction. In specific cases, the electric monopole interaction gives rise to the isomic shift (I.S.), which falls within the range of 0.1 ~ 0.6 mm s^-1^ (Figs. 2d and S6b), indicating that Fe predominantly exists in the form of Fe^3+^ in the LBZFO structure [28]. The magnetic dipole interaction also leads to resonance spectral line splitting known as magnetic hyfine splitting (*H*_*hf*_). Figures 2d and S6c demonstrate a significant reduction in *H*_*hf*_ upon initial Zr^4+^ doping into BaM due to substitution of non-magnetic Zr^4+^ ions for Fe^3+^ ions, weakening the superexchange interaction of Fe^3+^–O–Fe^3+^. However, subsequent replacement of rare-earth magnetic La^3+^ ions for non-magnetic Ba^2+^ ions would reinforce the superexchange effect, leading to gradual increase in *H*_*hf*_ values again (Fig. 2d) [29]. The electric quadrupole interaction causes quadrupole splitting of the resonance line referred to Q.S. (Figs. 2d and S6d). It can be seen that the Q.S. values remain nearly constant after La^3+^ ions incorporation into BaM lattice, suggesting minimal changes in system symmetry induced by doping behavior [30].

Moreover, first principles calculations were employed to estimate enthalpy for various sites where Zr^4+^ substitutes for Fe^3+^ in LBZFO sample. Figure 2e summarizes calculation results for five different structure models ranging from 0 to 3000 pm. It reveals that the crystalline structure with Zr^4+^ substituting for Fe^3+^ at 4f_2_ position has the minimum total enthalpy beyond 3000 pm, followed by 12k, 2a and 4f_1_ positions. According to the principle that lower energy levels contribute to increased stability in structures, which interprets the prominent rearrangement of Zr^4+^ substitution sites from 4f_1_ to 2a and 4f_2_ by La^3+^ doping in the perspective view of minimum energy principle. To sum up, Zr^4+^ ions initially tend to occupy the 12k and 4f_1_ sites of Fe^3+^ ions in BaM; while, they undergo a significant rearrangement of positions from 4f_1_ to 2a and 4f_2_ by La^3+^ doping for structure stability.

### Electromagnetic Response Behavior and Related Mechanisms

#### Magnetic Response Behavior and Mechanism of the LBZFO Samples

**Fig. 3:**
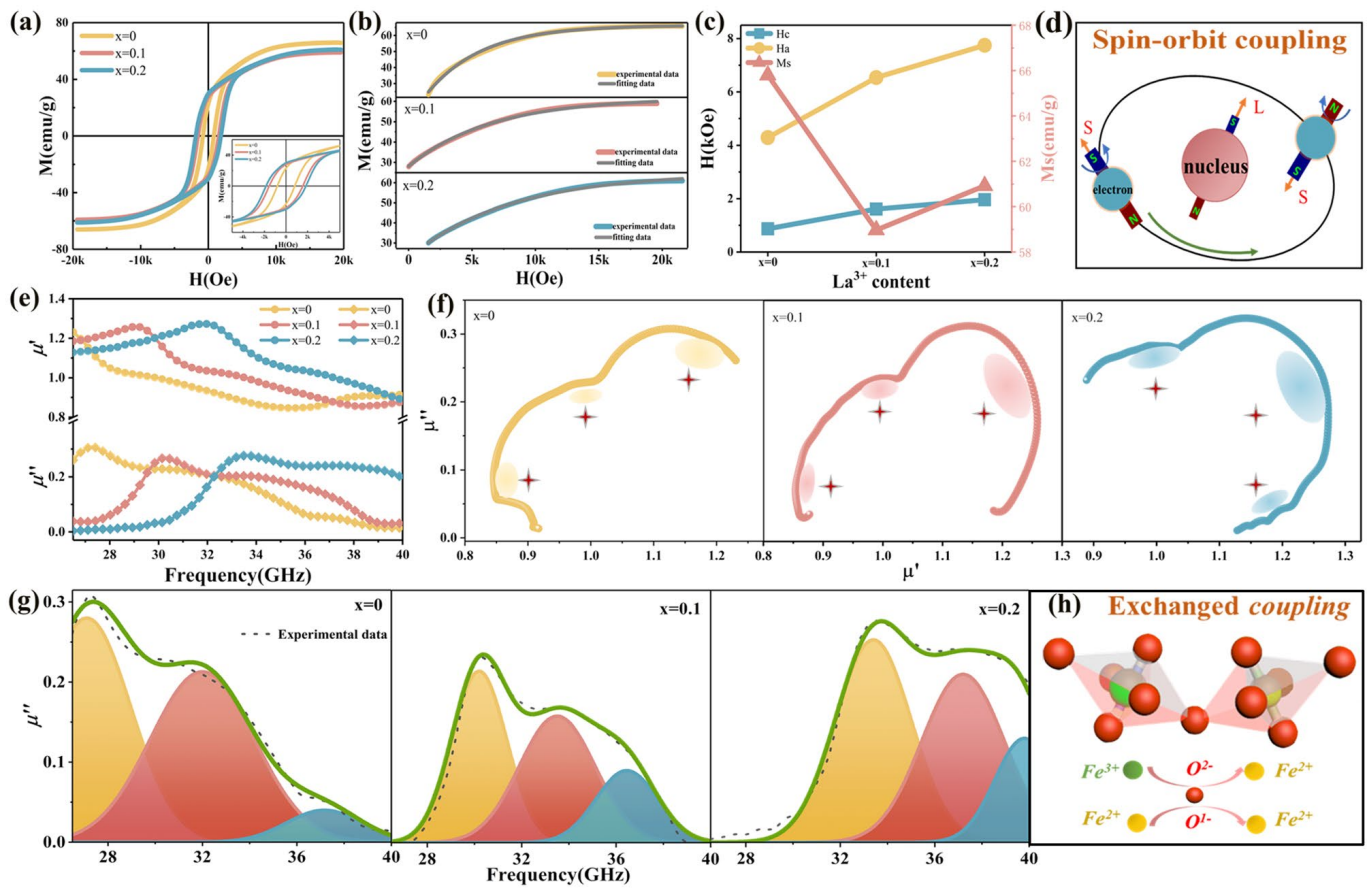
**a** Hysteresis loops, **b** fitting of the hysteresis loops by law of approach to saturation, **c** obtained *M*_*s*_, *H*_a,_ *H*_*c*_ values of the La_*x*_Ba_1-*x*_Zr_0.3_Fe_11.7_O_19_ samples with *x* = 0, 0.1, 0.2, **d** schematic diagram of spin orbit coupling effect, **e** real and imaginary parts of complex permeability, **f** Cole–Cole circles, **g** magnetic resonance peak-differentiating and imitating of the La_*x*_Ba_1-*x*_Zr_0.3_Fe_11.7_O_19_ samples with *x* = 0, 0.1, 0.2 and (**h**) schematic diagram of the exchange coupling between Fe^3+^ and Fe^2+^ ions

The hysteresis loops of the original BaM and the LBZFO samples are illustrated in Figs. S7 and 3a. All the specimens are nearly saturated when the external field reaches to 20 kOe. Figure 3b depicts the magnetization curves of the LBZFO samples fitted by the law of approach to saturation (LAS) based on the following formula:

3 $$M = M_{{\text{S}}} \left( {1 - {\text{A}}/H - {\text{B}}/H^{2} } \right) + \chi_{0} H$$

where A is the non-uniformity constant, B is a constant related to *H*_a_ and *χ*_0_ is the high magnetic susceptibility [31]. The fitting results of *M*_s_, *H*_a_ and the coercivity (*H*_*c*_) deduced from the hysteresis loops are summarized in Fig. 3c. It is found that the initial *M*_s_ value of original BaM is 62.84 emu g^-1^, which increases to a maximum value of 65.80 emu g^-1^ upon Zr^4+^ doping to form BaZr_0.3_Fe_11.7_O_19_. With further addition of La^3+^ ions, *M*_s_ firstly decreases to 58.97 emu g^-1^ with *x* = 0.1 and then increases slightly to 60.92 emu g^-1^ at *x* = 0.2. It has long been established that the saturation magnetization is collectively influenced by various factors, including the number, spin direction, magnetic moment of magnetic atoms/ions and the superexchange interaction, etc. [32]. In regard to BaM, the spin direction of Fe^3+^ ions at 12k, 2a, and 2b sites is upward; whereas, the spin direction of Fe^3+^ ions at 4f_1_ and 4f_2_ is opposite. The *M*_s_ is ultimately determined by the vector sum of spin-up magnetic ions [8, 12]. Therefore, the increment of *M*_s_ in BaZr_0.3_Fe_11.7_O_19_ is primarily attributed to the substitution of Fe^3+^ ions at down-spin 4f_1_ site by the non-magnetic Zr^4+^ ions. While with the further addition of La^3+^ ions, two significant effects occur simultaneously. Firstly, the transformation of Fe^3+^ into Fe^2+^ for electric neutrality would result in a distinct magnetic dilution with magnetic moment decreasing from 5 to 4 *µ*_B_ [33]. Secondly, the rare-earth La^3+^ ions improve the number of magnetic ions and superexchange interaction in BaM, which contributes to the enhancement of *M*_s_. Under the collaborative influence of multiple factors, the *M*_s_ consequently reveals a reduction firstly and then turns to a slight increase with La^3+^ ions content elevating (Fig. 3c). In regard to *H*_a_, it experiences a conspicuous growth from 9.47 to 11.74 kOe for an increased La^3+^ content from *x* = 0 to *x* = 0.2. This phenomenon can be attributed to two primary causes. One is that the rare-earth La^3+^ has a larger magnetocrystalline anisotropy constant than the substituted Ba^2+^ due to the strong spin–orbit coupling effect as illustrated in Fig. 3d [11]. The other one is ascribed to the transformation of Fe^3+^ to Fe^2+^, where Fe^2+^ possesses a higher magnetocrystalline anisotropy constant for partly frozen orbital angular momentum [34, 35]. As for *H*_*c*_, it increases from 0.87 kOe (*x* = 0) to the maximum value of 1.96 kOe (*x* = 0.2). Considering the fact that *H*_*c*_ is jointly determined by *H*_a_ and grain size, improving with increasing *H*_a_ or decreasing grain size [36]. By observation of Fig. 1b, the grain size hardly changes by La^3+^ ions doping. Therefore, the improvement of *H*_*c*_ with La^3+^ content increasing is dominantly contributed by the correspondingly enhanced *H*_a_.

Figures S8 and 3e display the real and imaginary parts of the complex permeability for the original BaM and LBZFO samples across a frequency spectrum of 26.5–40 GHz. Apparently, no magnetic resonance behavior occurs in the original BaM; whereas, all LBZFO samples display an asymmetric magnetic resonance phenomenon. Previous literature has reported that the natural resonance frequency of pure BaM lies approximately at 45 GHz [37], which exceeds the upper limit of our measuring capabilities. The substitution of non-magnetic Zr^4+^ ions for Fe^3+^ ions in BaM would lead to a decrease in *H*_a_ and thus reduces the natural resonance frequency to ~ 27 GHz with the constituent of BaZr_0.3_Fe_11.7_O_19_. After the additional La^3+^ ions doping, it can be observed that the resonance peak frequency gradually shifts toward the high-frequency direction to atmospheric window of 35 GHz. According to the expression of the natural resonance frequency:

4 $$f_{r} = \frac{\gamma }{2\pi } = 1.4gH_{a}$$

where $\gamma$ is gyromagnetic ratio, *g* is Landé factor. It is known that the resonance frequency is directly proportional to *H*_a_. The reinforced *H*_a_ by La^3+^ ions doping is hence the root cause for the increase in natural resonance frequency to approach the atmospheric window of 35 GHz.

The Cole–Cole plots of permeability are drawn in Fig. 3f to elucidate the multifaceted magnetic resonance relaxation occurring beneath the asymmetric resonance peaks. As a matter of fact, the permeability induced by resonance behavior should obey the equation as follows [38]:

5 $$\left( {\mu^{\prime \prime } } \right)^{2} + \left( {\mu^{\prime } } \right)^{2} = \left( {\frac{{B_{m} }}{{\mu_{0} H_{m} }}} \right)^{2}$$

where *µ*_0_ is permeability in free space, *H*_*m*_ and *B*_*m*_ are the amplitude of the magnetic field and the magnetic induction intensity, respectively. That is to say, each semicircle in *µ*"-*µ*' curves correspond to a magnetic resonance behavior. The presence of three distinct semicircles in the LBZFO samples strongly suggests the existence of three predominant resonance loss mechanisms within the specified frequency range. To gain a deeper understanding of the multiple magnetic resonance behavior, the asymmetric resonance peaks are further analyzed using the Peakfit software as depicted in Fig. 3g. Intriguingly, they all could be resolved into three distinct peaks at the frequency range of 26.5–40 GHz. The frequencies of the three resolved peaks are (27.10, 32.00, and 37.16 GHz), (30.20, 33.50, and 36.45 GHz) and (33.40, 37.20, and 39.80 GHz) when *x* = 0, 0.1, and 0.2, respectively. Actually, the *g* values of Fe^3+^ and Fe^2+^ are 2.0 and 3.54, separately. The exchange coupling between these two ion species, as illustrated in Fig. 3h, can lead to the formation of new *g* factors that fall within the range of 2.0 to 3.54 [12, 39]. Based on the formula of natural resonance frequency, the *g* values for the resolved peaks are determined to be (2.04, 2.41, 2.80), (1.90, 2.11, 2.29), and (2.03, 2.26, 2.42), respectively. This analysis confirms that, apart from the natural resonance originating from the intrinsic Fe^3+^ ions with *g* values around 2.0 (1.90 ~ 2.04), the exchange coupling between Fe^3+^ and Fe^2+^ ions with *g* values ranging from 2.11 to 2.80 enhances the formation of impressive magnetic loss peaks within the higher frequency range. Furthermore, it is worth emphasizing that the exchange coupling effect by the dual high-valance ions doping is more pronounced around the atmospheric window of 35 GHz when compared to the series doped solely with Zr^4+^ ions in Fig. S9, Ti^4+^ ions in Fig. S10 or Nb^5+^ ions in Fig. S11. These results unambiguously demonstrate the beneficial impact of the dual high-valance ion doping strategy in enhancing the magnetic loss capacity within the target frequency range. In conclusion, the contrasting impact of La^3+^ and Zr^4+^ ions on *H*_a_ allows for precise tuning of inherent resonance to the desired atmospheric window of 35 GHz. Meanwhile, the co-substitution of the two high-valence ions can effectively intensify the exchange coupling effect between Fe^3+^ and Fe^2+^ to gain wide and strong magnetic loss.

#### Dielectric Response Behavior and Mechanism of the LBZFO Samples

**Fig. 4:**
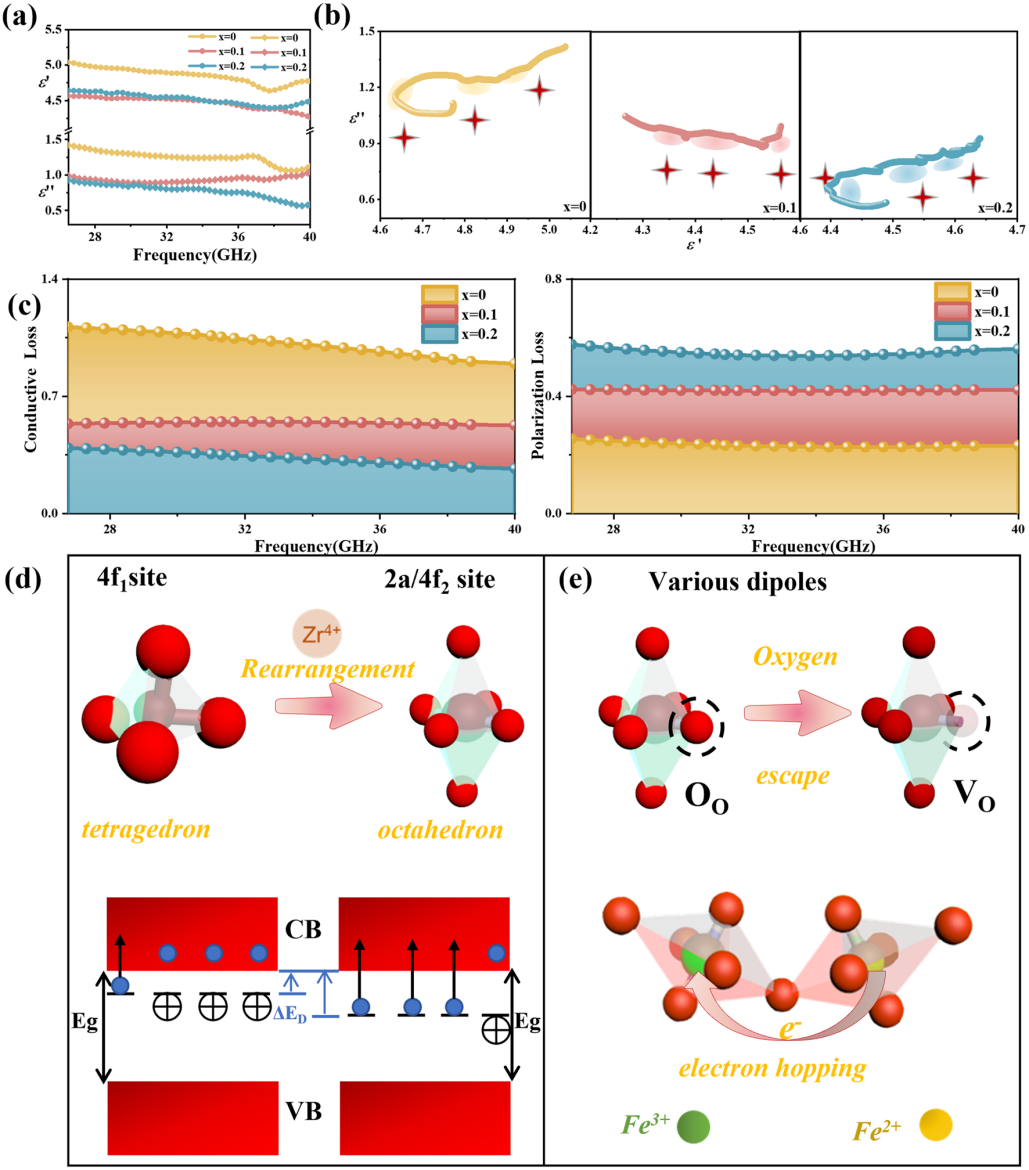
**a** The real and imaginary parts of complex permittivity, **b** Cole–Cole circles, **c** conduction loss/polarization loss portion obtained by least square fitting of the La_*x*_Ba_1-*x*_Zr_0.3_Fe_11.7_O_19_ samples with *x* = 0, 0.1, 0.2, **d** schematic diagram of diminished conduction loss mechanism and **e** schematic diagram of various dipoles in the La^3+^ and Zr^4+^ co-doped barium ferrite

The complex permittivity values of the original BaM and LBZFO samples plotted against frequency are clearly visible in Figs. S8b and 4a. Upon initial doping with Zr^4+^ ions, both real part (*ε*’) and imaginary part (*ε*") of the dielectric constant reveal an evident enhancement. Subsequently, with the introduction of additional La^3+^ ions, these values exhibit a notable decrease. While with La^3+^ content further rising from *x* = 0.1 to *x* = 0.2, the permittivity values remain relatively stable with a slight decrease observed only in the high-frequency range of *ε*". It is universally acknowledged that the permittivity is comprised by two distinct behaviors: conduction and polarization [40]. In order to gain a comprehensive understanding for the alteration mechanisms of permittivity, the Cole–Cole curves are plotted with *ε*’ and *ε*" as the horizontal and longitudinal coordinates as shown in Fig. 4b firstly. According to the Debye equation expression as follows [41–43]:

6 $$\left( {\varepsilon^{\prime } - \frac{{\varepsilon_{s} + \varepsilon_{\infty } }}{2}} \right)^{2} + \left( {\varepsilon^{\prime \prime } } \right)^{2} = \left( {\frac{{\varepsilon_{s} + \varepsilon_{\infty } }}{2}} \right)^{2}$$

where ${\varepsilon}_{\infty}$ and *ε*_s_ represent the relative permittivity under infinite frequency and static permittivity. The Debye relaxing process is consequently represented as a circle in the Cole–Cole diagram. From Fig. 4b, there are three obvious semicircles in the LBZFO samples, which indicates the existence of abundant polarization relaxations. Besides, the *ε*"-*ε*' curves also display sections of partial straight lines, demonstrating that conduction loss also contributes to dielectric loss to some extent [44]. To quantitatively assess the contributions of conductive and polarization loss, the least square method was employed based on variations in the Debye equation [45]. As depicted in Fig. 4c, the conduction loss of the LBZFO samples gradually decreases with increasing La^3+^ doping proportion; while, the polarization loss exhibits an opposite increasing trend. In fact, the diminished conduction loss probably originates from the rearrangement of Zr^4+^ ions from tetrahedral 4f_1_ site to octahedral 2a/4f_2_ sites after La^3+^ doping, which is probably owing to the lower impurity energy level provided by Zr^4+^ substitution in the higher symmetry octahedron (Fig. 4d) [12]. On the other side, as the portions of Fe^2+^ ions and oxygen vacancy increase gradually with the La^3+^ doping ratio increasing based on the aforementioned XPS results in Fig. 1d, e, numerous Fe^3+^/Fe^2+^, Zr^4+^/VӦ, La^3+^/VӦ dipoles are thus generated in the LBZFO samples as illustrated in Fig. 4e for efficiently reinforcing the polarization loss [8, 12]. By reason of the foregoing, the additional incorporation of La^3+^ ions is expected to meticulously regulate the weight coefficient of conduction loss and polarization loss for achieving a suitable dielectric loss performance.

### Millimeter-Wave Absorption Performance and Radar Stealth Simulation

#### Evaluation of Millimeter-Wave Absorption Performance for the LBZFO Samples

**Fig. 5:**
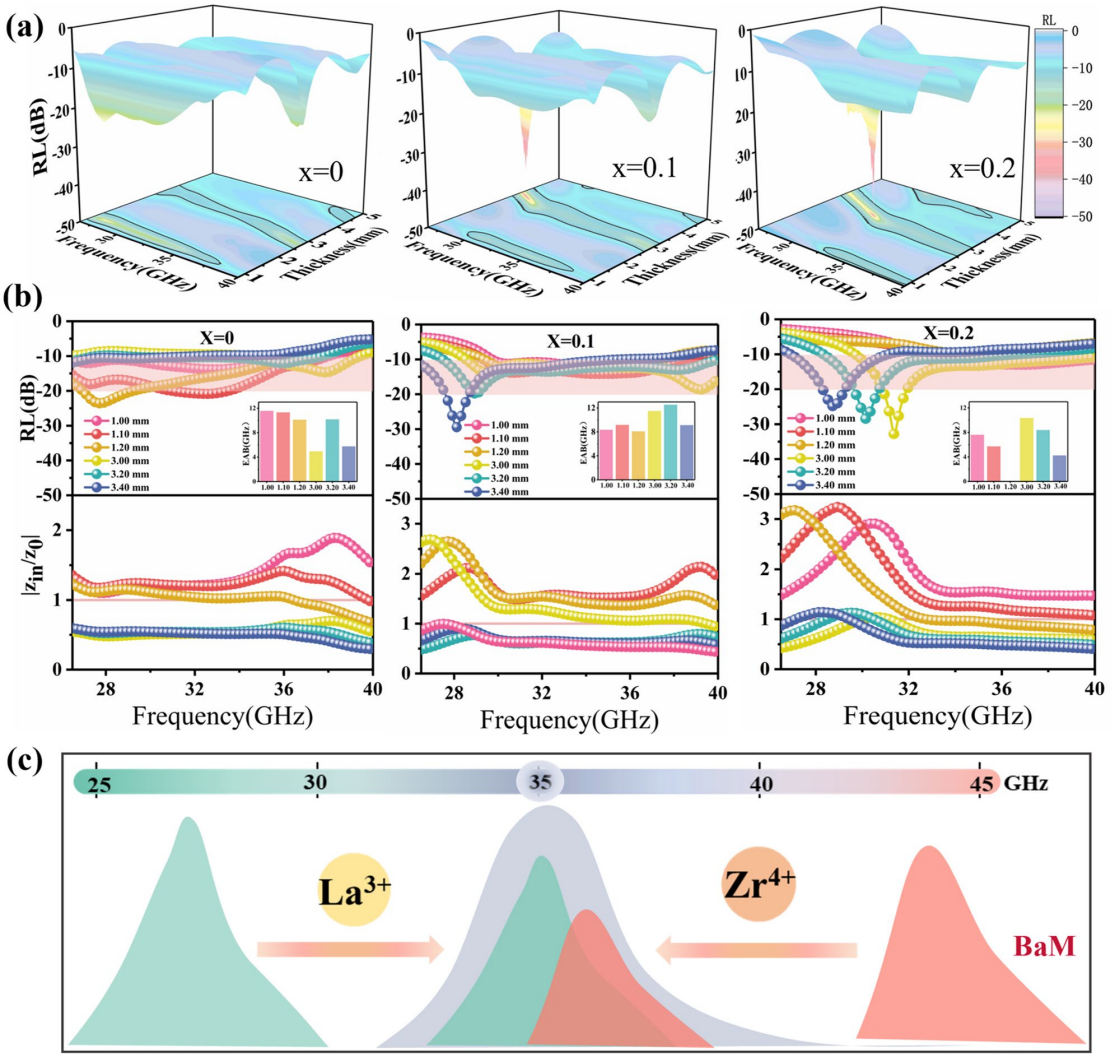
**a** 3D reflection loss values versus frequency and thickness, **b** 2D reflection loss and impedance matching values of the La_*x*_Ba_1-*x*_Zr_0.3_Fe_11.7_O_19_ samples with *x* = 0, 0.1, 0.2 and **c** schematic diagram illustrating the effects of La^3+^ and Zr^4+^ ions on natural resonance frequency

The microwave absorption performance is usually evaluated by the reflection loss (RL) values calculated by transmission line theory based on the following equations [46–53]:

7 $${\text{RL}} = 20\log \left| {\frac{{Z_{{{\text{in}}}} - Z_{0} }}{{Z_{{{\text{in}}}} - Z_{0} }}} \right|$$

8 $$Z_{{{\text{in}}}} = Z_{0} \sqrt {\frac{{\mu_{r} }}{{\varepsilon_{r} }}} \tanh \left[ {j\frac{2\pi fd}{c}\sqrt {\mu_{r} \varepsilon_{r} } } \right]$$

where *Z*_in_ is the input impedance of absorber, *Z*_0_ is the characteristic impedance of free space,* ε*_*r*_ is complex permittivity (*ε*_*r*_ = *ε*'-*jε*"), *µ*_*r*_ is the permeability (*µ*_*r*_ = *µ*'-*jµ*") of the ferrites, *f* is wave frequency, *d* is the thickness of the absorption layer and *c* is the velocity of light. Figure S12 displays that the original BaM is not equipped with absorption capacity in the range of 26.5 ~ 40 GHz owing to the deficiency in magnetic and dielectric loss. However, as shown in Fig. 5a, the effective absorption band can be observed in the matching thickness range of *n* = 1, *n* = 3 and *n* = 5 by Zr^4+^ and La^3+^ ions co-doping, and the RL has a smaller value in the matching thickness range of *n* = 3 with the increase in La^3+^ doping ratio. Specifically, Fig. 5b shows the two-dimensional RL curves of the LBZFO samples at various thicknesses. Among the rest, the corresponding EAB and impedance matching values are also merged, and the pink areas highlight the effective absorption range where the RL values are below - 10 dB, meaning the microwave absorption exceeds 90%. It demonstrates that the LBZFO sample with *x* = 0.1 exhibits the optimal absorption performance around the atmospheric window of 35 GHz. The effective absorption range (RL < - 10 dB) covers 29.4 ~ 37.4 GHz (8.0 GHz) under the first order of matching thickness (*n* = 1) at 1.2 mm. Intriguingly, it significantly extends to the widest EAB range of 27.5 ~ 40.0 + GHz (12.5 + GHz) under the second order of matching thickness (*n* = 3) at 3.2 mm, accompanied by an impressively low RL_min_ value of -19.8 dB. The millimeter-wave absorption properties around the atmospheric window of 35 GHz are evidently superior than the Zr^4+^, Ti^4+^ or Nb^5+^ ions solely doped series (Figs. S13-S15), which is attributed to the impressively modified input impedance matching on the grounds of *Z*_in_/*Z*_0_ values in Fig. 5b. This is dominantly contributed by the precise regulation of natural resonance peak for approaching 35 GHz via the opposite effect of La^3+^ and Zr^4+^ ions on *H*_a_, strengthening the magnetic loss capacity in the corresponding frequency band as presented in Fig. 5c. Besides, the enhancive portion of polarization loss to conduction loss revealed in Fig. 4c is also believed to exert a positive effect on ameliorating the impedance matching to broaden the absorption bandwidth [54–57]. As a consequence, the co-incorporation of La^3+^ and Zr^4+^ ions into BaM serves as a highly competitive approach for the development of exceptional millimeter-wave absorbers, specifically designed for the 35 GHz atmospheric window.

#### Near-Field and Far-Field Simulation Based on the LBZFO Samples

**Fig. 6:**
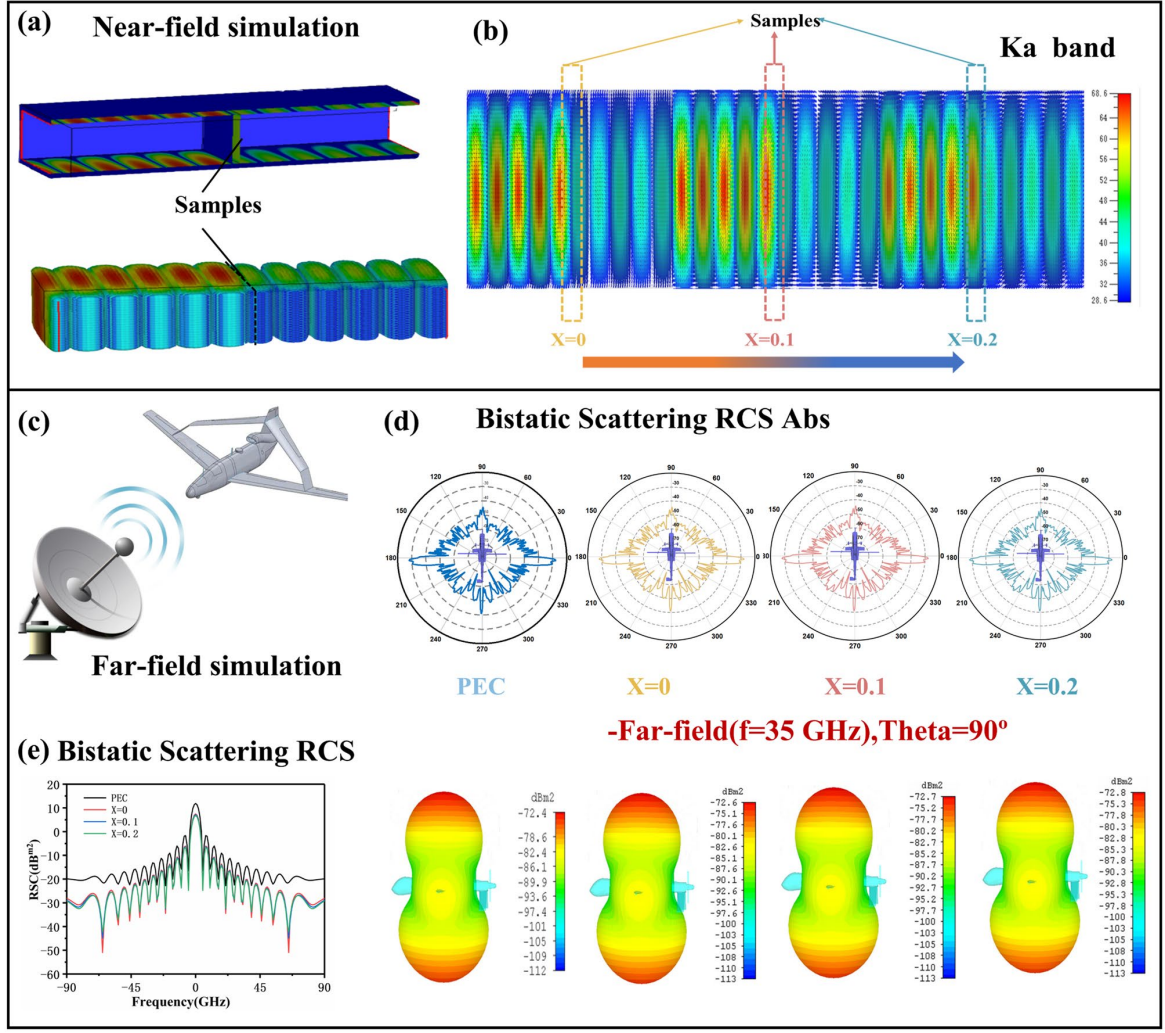
**a** Model diagram and **b** results diagram of near-field simulation, **c** model diagram and **d** results diagram of far-field simulation and **e** RSC values in the range of 0° ~ 360° for the La_*x*_Ba_1-*x*_Zr_0.3_Fe_11.7_O_19_ samples with *x* = 0, 0.1, 0.2

CST Studio Suite has been utilized to meticulously simulate the propagation characteristics of millimeter-waves through LBZFO samples, both in the near-field and far-field environments. The near-field simulation diagram is illustrated vividly in Fig. 6a, providing a clear visualization of the wave propagation. The results obtained from these simulations are presented in Fig. 6b, clearly showing the significant reduction in the intensities of millimeter-waves after passing through the LBZFO samples. This observation verifies the remarkable millimeter-wave absorption capacity of the elaborately designed LBZFO samples. In addition, as shown in Fig. 6c, a simplified Predator II UAV model with a length of 0.82 m and a width of 1.34 m is applied for the far-field simulation, where the atmospheric window center of 35 GHz is chosen as the field monitoring frequency and the open boundary is roundly set in all directions. Figure 6d shows the RSC values of the UAV model from 0° to 360° before/after coating the LBZFO samples with thickness of 1 mm. Evidently, the RCS values diminish effectively at omnidirectional angles with the usage of the LBZFO coatings, which can be more clearly observed through the scattered signals of three-dimensional radar waves as shown in Fig. 6e. Besides, the near-field and far-field simulations have also been conducted for the Zr^4+^, Ti^4+^ and Nb^5+^ ions solely doped series as seen in Figs. S16-S19. A comparative analysis of the results reveals that the LBZFO coatings contribute significantly to reduce the radar detection probability for military equipment across a wide range of angles. This finding holds tremendous promise of the LBZFO coatings for applications in the field of millimeter-wave absorption.

## Conclusions

The La^3+^–Zr^4+^ ions are successfully incorporated into BaM through a sol–gel process, which allows for an intentional manipulation on multi-magnetic resonance behavior. The La^3+^ and Zr^4+^ ions are verified to substitute for Ba^2+^ and Fe^3+^ ions of BaM, respectively. With further refinement, it is found that Zr^4+^ ions occupy Fe^3+^ ions at 12k and 4f_1_ sites initially. However, upon additional doping of La^3+^ ions, a profound rearrangement of Zr^4+^ ions occurred from the 4f_1_ site to the 2a and 4f_2_ sites for structural stability. This rearrangement of Zr^4+^ ions from a tetrahedron to an octahedron configuration with higher symmetry leads to a lower impurity energy level, making the portion of polarization/conduction loss increase gradually with the rising La^3+^ content. Moreover, the contrasting effects of La^3+^ and Zr^4+^ ions in *H*_a_ contribute to the regulation of multiple magnetic resonances, which emerge within the atmospheric window at approximately 35 GHz. The dual high-valance ions doping also intensifies the exchange coupling effect between Fe^3+^ and Fe^2+^ ions, boosting the magnetic loss capacity in the respective frequency range. Eventually, due to the substantial improvement of input impedance matching, the LBZFO samples possess an extremely broad EAB of 12.5 + GHz around 35 GHz atmospheric window and effectively diminish the RCS values at omnidirectional angles, indicating the significant potential in military stealth and electromagnetic pollution prevention applications.

## Supplementary Information

Below is the link to the electronic supplementary material.

Supplementary file1 (PDF 1701 kb)

## References

[1] Z. Zhao, Y. Qing, L. Kong, H. Xu, X. Fan et al., Advancements in microwave absorption motivated by interdisciplinary research. Adv. Mater. **36**, 2304182 (2024). 10.1002/adma.202304182 10.1002/adma.202304182 37870274

[2] G. Giribaldi, L. Colombo, P. Simeoni, M. Rinaldi, Compact and wideband nanoacoustic pass-band filters for future 5G and 6G cellular radios. Nat. Commun. **15**, 304 (2024). 10.1038/s41467-023-44038-9 38182572 10.1038/s41467-023-44038-9 PMC10770411

[3] J. Cheng, H. Zhang, M. Ning, H. Raza, D. Zhang et al., Emerging materials and designs for low- and multi-band electromagnetic wave absorbers: The search for dielectric and magnetic synergy? Adv. Funct. Mater. **32**, 2200123 (2022). 10.1002/adfm.202200123

[4] C. Xue, W. Wu, Y. Yang, J. Zhou, L. Ding et al., Carbon nanotube diodes operating at frequencies of over 50 GHz. Small **19**, 2207628 (2023). 10.1002/smll.202207628 10.1002/smll.202207628 36808872

[5] C. Liu, Y. Zhang, Y. Tang, Z. Wang, H. Tang et al., Excellent absorption properties of BaFe_12-__*x*_Nb_*x*_O_19_ controlled by multi-resonance permeability, enhanced permittivity, and the order of matching thickness. Phys. Chem. Chem. Phys. **19**, 21893–21903 (2017). 10.1039/C7CP03014B 28787052 10.1039/c7cp03014b

[6] M. Yuan, B. Zhao, C. Yang, K. Pei, L. Wang et al., Remarkable magnetic exchange coupling via constructing Bi-magnetic interface for broadband lower-frequency microwave absorption. Adv. Funct. Mater. **32**, 2203161 (2022). 10.1002/adfm.202203161

[7] F. He, W. Zhao, L. Cao, Z. Liu, L. Sun et al., The ordered mesoporous Barium ferrite compounded with nitrogen-doped reduced graphene oxide for microwave absorption materials. Small **19**, e2205644 (2023). 10.1002/smll.202205644 37078836 10.1002/smll.202205644

[8] C. Liu, Y. Zhang, Y. Tang, Z. Wang, N. Ma et al., The tunable magnetic and microwave absorption properties of the Nb^5+^–Ni^2+^ Co-doped M-type Barium ferrite. J. Mater. Chem. C **5**, 3461–3472 (2017). 10.1039/C7TC00393E

[9] J. Li, Y. Hong, S. He, W. Li, H. Bai et al., A neutron diffraction investigation of high valent doped Barium ferrite with wideband tunable microwave absorption. J. Adv. Ceram. **11**, 263–272 (2022). 10.1007/s40145-021-0529-3

[10] C. Liu, Y. Hao, S. Zheng, G. Fang, J. Li et al., Abating dopant competition between dual high-valence ions in single-phased Barium ferrite towards ultra-broad microwave absorption. J. Mater. Chem. C **11**, 15500–15511 (2023). 10.1039/D3TC03246A

[11] B.Y. Wang, T.C. Wang, Y.T. Hsu, M. Osada, K. Lee et al., Effects of rare-earth magnetism on the superconducting upper critical field in infinite-layer nickelates. Sci. Adv. **9**, eadf6655 (2023). 10.1126/sciadv.adf6655 37196089 10.1126/sciadv.adf6655 PMC10191431

[12] C. Liu, Q. Xu, Y. Tang, Z. Wang, R. Ma et al., Zr^4+^ doping-controlled permittivity and permeability of BaFe_12-__*x*_Zr_*x*_O_19_ and the extraordinary EM absorption power in the millimeter wavelength frequency range. J. Mater. Chem. C **4**, 9532–9543 (2016). 10.1039/C6TC03430F

[13] H. Shang, J. Wang, Q. Liu, Synthesis and characterization of nanocrystalline BaFe_12_O_19_ obtained by using glucose as a fuel. Mater. Sci. Eng. A **456**, 130–132 (2007). 10.1016/j.msea.2006.12.011

[14] S.P. Keerthana, R. Yuvakkumar, G. Ravi, A.G. Al-Sehemi, D. Velauthapillai, Synthesis of pure and lanthanum-doped Barium ferrite nanoparticles for efficient removal of toxic pollutants. J. Hazard. Mater. **424**, 127604 (2022). 10.1016/j.jhazmat.2021.127604 34763285 10.1016/j.jhazmat.2021.127604

[15] X. Wang, B. Wang, S. Wei, Y. Wang, Y. Liang et al., Tunable magnetic and microwave absorption properties of Barium ferrite particles by site-selective Co^2+^-Zr^4+^ Co-doping. J. Alloys Compd. **960**, 170777 (2023). 10.1016/j.jallcom.2023.170777

[16] A.M. Youssef, S.M. Yakout, S.M. Mousa, High relative permittivity and excellent dye photo-elimination: pure and (Zr^4+^, Y^3+^, Sb^5+^) multi-doped anatase TiO_2_ structure. Opt. Mater. **135**, 113261 (2023). 10.1016/j.optmat.2022.113261

[17] J. He, Y. Liu, Y. Huang, H. Li, Y. Zou et al., Fe^2+^-induced *in situ* intercalation and cation exsolution of Co_80_Fe_20_(OH)(OCH_3_) with rich vacancies for boosting oxygen evolution reaction. Adv. Funct. Mater. **31**, 2009245 (2021). 10.1002/adfm.202009245

[18] C. Liu, Y. Zhang, Y. Zhang, G. Fang, X. Zhao et al., Multiple nature resonance behavior of BaFe_*x*_TiO19 controlled by Fe/Ba ratio and its regulation on microwave absorption properties. J. Alloys Compd. **773**, 730–738 (2019). 10.1016/j.jallcom.2018.09.278

[19] B. Xiao, C. Liu, D. Pan, R. Hu, T. Sun et al., A solid solution-based millimeter-wave absorber exhibiting highly efficient absorbing capability and ultrabroad bandwidth simultaneously *via* a multi-elemental co-doping strategy. J. Mater. Chem. C **10**, 1381–1393 (2022). 10.1039/D1TC05078H

[20] R. Jasrotia, V. Pratap Singh, R. Kumar, K. Singha, M. Chandel et al., Analysis of Cd^2+^ and In^3+^ ions doping on microstructure, optical, magnetic and m o¨ ssbauer spectral properties of sol-gel synthesized BaM hexagonal ferrite based nanomaterials. Results Phys. **12**, 1933–1941 (2019). 10.1016/j.rinp.2019.01.088

[21] Z. Durmus, H. Sozeri, M.S. Toprak, A. Baykal, The effect of condensation on the morphology and magnetic properties of modified Barium hexaferrite (BaFe_12_O_19_). Nano-Micro Lett. **3**, 108–114 (2011). 10.1007/BF03353659

[22] C. Liu, Z. Chen, X. Xiang, G. Fang, Z. Wang et al., Determining the actual composition of Nb^5+^–Ni^2+^ codoped Barium ferrites to controllably regulate the microwave absorbing properties. J. Phys. Chem. C **126**, 21800–21809 (2022). 10.1021/acs.jpcc.2c06747

[23] W. Yuan, L. Cheng, T. Xia, Y. Chen, Q. Long et al., Effect of Fe doping on the lattice structure, microscopic morphology and microwave absorption properties of LaCo_1-x_Fe_x_O_3_. J. Alloys Compd. **926**, 166839 (2022). 10.1016/j.jallcom.2022.166839

[24] W.-Y. Zhao, P. Wei, H.-B. Cheng, X.-F. Tang, Q.-J. Zhang, FTIR spectra, lattice shrinkage, and magnetic properties of CoTi-substituted M-type Barium hexaferrite nanoparticles. J. Am. Ceram. Soc. **90**, 2095–2103 (2007). 10.1111/j.1551-2916.2007.01690.x

[25] Z. Zhang, J. Li, J. Qian, Z. Li, L. Jia et al., Significant change of metal cations in geometric sites by magnetic-field annealing FeCo_2_O_4_ for enhanced oxygen catalytic activity. Small **18**, e2104248 (2022). 10.1002/smll.202104248 34877765 10.1002/smll.202104248

[26] S. Lu, Y. Liu, Q. Yin, J. Chen, J. Wu et al., Investigation of crystal structure, Raman spectroscopy and magnetic properties of La-Zn substituted oriented M-type hexagonal Barium ferrites. Mater. Res. Bull. **172**, 112640 (2024). 10.1016/j.materresbull.2023.112640

[27] Y. Shao, F. Huang, X. Xu, S. Yan, C. Yang et al., Multi-susceptible single-phase BaAl_*x*_Fe_12–__*x*_O_19_ ceramics with both improved magnetic and ferroelectric properties. Appl. Phys. Lett. **114**, 242902 (2019). 10.1063/1.5094069

[28] M.I. Ariëns, L.G.A. van de Water, A.I. Dugulan, E. Brück, E.J.M. Hensen, Copper promotion of chromium-doped iron oxide water-gas shift catalysts under industrially relevant conditions. J. Catal. **405**, 391–403 (2022). 10.1016/j.jcat.2021.12.013

[29] Z.H. Hua, S.Z. Li, Z.D. Han, D.H. Wang, M. Lu et al., The effect of La–Zn substitution on the microstructure and magnetic properties of Barium ferrites. Mater. Sci. Eng. A **448**, 326–329 (2007). 10.1016/j.msea.2006.11.153

[30] C. Wang, Y. Liu, Z. Jia, W. Zhao, G. Wu, Multicomponent nanoparticles synergistic one-dimensional nanofibers as heterostructure absorbers for tunable and efficient microwave absorption. Nano-Micro Lett. **15**, 13 (2022). 10.1007/s40820-022-00986-3 10.1007/s40820-022-00986-3 PMC9755410 36520259

[31] P. Wu, X. Kong, Y. Feng, W. Ding, Z. Sheng et al., Phase engineering on amorphous/crystalline *γ*-Fe_2_O_3_ nanosheets for boosting dielectric loss and high-performance microwave absorption. Adv. Funct. Mater. **34**, 2311983 (2024). 10.1002/adfm.202311983

[32] Q. Jin, Q. Zhang, H. Bai, A. Huon, T. Charlton et al., Emergent magnetic states and tunable exchange bias at 3d nitride heterointerfaces. Adv. Mater. **35**, e2208221 (2023). 10.1002/adma.202208221 36300813 10.1002/adma.202208221

[33] S. Kumar, M. Kumar Manglam, S. Supriya, H. Kumar Satyapal, R. Kumar Singh et al., Lattice strain mediated dielectric and magnetic properties in La doped Barium hexaferrite. J. Magn. Magn. Mater. **473**, 312–319 (2019). 10.1016/j.jmmm.2018.10.085

[34] H. Koizumi, J.-I. Inoue, H. Yanagihara, Magnetic anisotropy and orbital angular momentum in the orbital ferrimagnet CoMnO_3_. Phys. Rev. B **100**, 224425 (2019). 10.1103/physrevb.100.224425

[35] M. He, J. Hu, H. Yan, X. Zhong, Y. Zhang et al., Shape anisotropic chain-like coni/polydimethylsiloxane composite films with excellent low-frequency microwave absorption and high thermal conductivity. Adv. Fucnt. Mater. **10**, 2316691 (2024). 10.1002/adfm.202316691

[36] E.A. Gorbachev, L.A. Trusov, A.E. Sleptsova, E.S. Kozlyakova, L.N. Alyabyeva et al., Hexaferrite materials displaying ultra-high coercivity and sub-terahertz ferromagnetic resonance frequencies. Mater. Today **32**, 13–18 (2020). 10.1016/j.mattod.2019.05.020

[37] Y. Ren, H. Yan, X. Li, S. Lv, H. Zhu et al., Enhanced saturation magnetization and microwave absorption magnetic properties of Mn-Co-Zr substituted BaM ferrite. J. Alloys Compd. **693**, 1257–1260 (2017). 10.1016/j.jallcom.2016.10.075

[38] F. Wang, Y. Liu, R. Feng, X. Wang, X. Han et al., A “win–win” strategy to modify Co/C foam with carbon microspheres for enhanced dielectric loss and microwave absorption characteristics. Small **19**, 2303597 (2023). 10.1002/smll.202303597 10.1002/smll.202303597 37528502

[39] A. Pacewicz, J. Krupka, J.H. Mikkelsen, A. Lynnyk, B. Salski, Accurate measurements of the ferromagnetic resonance linewidth of single crystal BaM hexaferrite spheres employing magnetic plasmon resonance theory. J. Magn. Magn. Mater. **580**, 170902 (2023). 10.1016/j.jmmm.2023.170902

[40] X. Guan, S. Tan, L. Wang, Y. Zhao, G. Ji, Electronic modulation strategy for mass-producible ultrastrong multifunctional biomass-based fiber aerogel devices: interfacial bridging. ACS Nano **17**, 20525–20536 (2023). 10.1021/acsnano.3c07300 37815393 10.1021/acsnano.3c07300

[41] M. Salari, S.M. Taromsari, S. Habibpour, H.H. Shi, M. Hamidinejad et al., The intersection of computational design and wearable-optimized electrospun structural nanohybrids for electromagnetic absorption. Adv. Funct. Mater. **34**, 2309528 (2024). 10.1002/adfm.202309528

[42] H. Luo, B. Ma, F. Chen, S. Zhang, Y. Xiong et al., Bimetallic oxalate rod-derived NiFe/Fe_3_O_4_@C composites with tunable magneto-dielectric properties for high-performance microwave absorption. J. Phys. Chem. C **125**, 24540–24549 (2021). 10.1021/acs.jpcc.1c04386

[43] F. Chen, H. Luo, Y. Cheng, R. Guo, W. Yang et al., Nickel/Nickel phosphide composite embedded in N-doped carbon with tunable electromagnetic properties toward high-efficiency microwave absorption. Compos. Part A Appl. Sci. Manuf. **140**, 106141 (2021). 10.1016/j.compositesa.2020.106141

[44] Y. Zhao, Z. Lin, L. Huang, Z. Meng, H. Yu et al., Simultaneous optimization of conduction and polarization losses in CNT@NiCo compounds for superior electromagnetic wave absorption. J. Mater. Sci. Technol. **166**, 34–46 (2023). 10.1016/j.jmst.2023.04.045

[45] F. Long, Y. Xu, X. Li, L. Ren, J. Shi et al., Comparative study of recursive least squares with variable forgetting factor applied in AC loss measurement. Phys. Scr. **99**, 015523 (2024). 10.1088/1402-4896/ad1703

[46] Y. Zou, J. Lin, W. Zhou, M. Yu, J. Deng et al., Coexistence of high magnetic and dielectric properties in Ni-Zr Co-doped Barium hexaferrites. J. Alloys Compd. **907**, 164516 (2022). 10.1016/j.jallcom.2022.164516

[47] M. Zhou, S. Tan, J. Wang, Y. Wu, L. Liang et al., “three-in-one” multi-scale structural design of carbon fiber-based composites for personal electromagnetic protection and thermal management. Nano-Micro Lett. **15**, 176 (2023). 10.1007/s40820-023-01144-z 10.1007/s40820-023-01144-z PMC10333170 37428269

[48] J. Hong, A. Bhardwaj, Y. Namgung, H. Bae, S.-J. Song, Evaluation of the effects of nanocatalyst infiltration on the SOFC performance and electrode reaction kinetics using the transmission line model. J. Mater. Chem. A **8**, 23473–23487 (2020). 10.1039/D0TA07166H

[49] X. Chen, S. Guo, S. Tan, J. Ma, T. Xu et al., An environmentally friendly chitosan-derived VO_2_/carbon aerogel for radar infrared compatible stealth. Carbon **213**, 118313 (2023). 10.1016/j.carbon.2023.118313

[50] X. Sun, Y. Li, Y. Huang, Y. Cheng, S. Wang et al., Achieving super broadband electromagnetic absorption by optimizing impedance match of rGO sponge metamaterials. Adv. Funct. Mater. **32**, 2107508 (2022). 10.1002/adfm.202107508

[51] F. Chen, S. Zhang, B. Ma, Y. Xiong, H. Luo et al., Bimetallic CoFe-MOF@Ti_3_C_2_T_x_ MXene derived composites for broadband microwave absorption. Chem. Eng. J. **431**, 134007 (2022). 10.1016/j.cej.2021.134007

[52] H. Luo, B. Ma, F. Chen, S. Zhang, X. Wang et al., Construction of hollow core-shelled nitrogen-doped carbon-coated yttrium aluminum garnet composites toward efficient microwave absorption. J. Colloid Interface Sci. **622**, 181–191 (2022). 10.1016/j.jcis.2022.04.054 35490621 10.1016/j.jcis.2022.04.054

[53] B. Ma, F. Chen, Y. Cheng, X. Wang, S. Yan et al., Ti_3_C_2_T_x_ MXene@NiFe layered double hydroxide derived multiple interfacial composites with efficient microwave absorption. J. Alloys Compd. **936**, 168162 (2023). 10.1016/j.jallcom.2022.168162

[54] G. Fang, T. He, X. Hu, X. Yang, S. Zheng et al., Bionic octopus structure Inspired Stress-Driven reconfigurable microwave absorption and multifunctional compatibility in infrared stealth and De-icing. Chem. Eng. J. **467**, 143266 (2023). 10.1016/j.cej.2023.143266

[55] G. Fang, C. Liu, X. Wei, Q. Cai, C. Chen et al., Determining the preferable polarization loss for magnetoelectric microwave absorbers by strategy of controllably regulating defects. Chem. Eng. J. **463**, 142440 (2023). 10.1016/j.cej.2023.142440

[56] X. Zhong, M. He, C. Zhang, Y. Guo, J. Hu et al., Heterostructured BN@Co-C@C endowing polyester composites excellent thermal conductivity and microwave absorption at C band. Adv. Funct. Mater. (2024). 10.1002/adfm.202313544

[57] J. Xiao, B. Zhan, M. He, X. Qi, X. Gong et al., Interfacial polarization loss improvement induced by the hollow engineering of necklace-like PAN/carbon nanofibers for boosted microwave absorption. Adv. Funct. Mater. (2024). 10.1002/adfm.202316722

