# Supplementary material for: Achieving Ultra-Broad Microwave Absorption Bandwidth Around Millimeter-Wave Atmospheric Window Through an Intentional Manipulation on Multi-Magnetic Resonance Behavior
Source: Nanomicro Lett. 2024 Apr 22;16:176. doi: 10.1007/s40820-024-01395-4 (PMC11035528; doi:10.1007/s40820-024-01395-4)
Supplement: Supplementary file 1 — Supplementary file1 (PDF 1701 kb) [file 40820_2024_1395_MOESM1_ESM.pdf]

Supporting Information for

Achieving Ultra-Broad Microwave Absorption Bandwidth around Millimeter-Wave Atmospheric Window through an Intentional Manipulation on Multi-Magnetic Resonance Behaviour

Chuyang Liu[1], Lu Xu[1], Xueyu Xiang[1], Yujing Zhang[2,*], Li Zhou[1], Bo Ouyang[3,*], Fan Wu[4], Dong-Hyun Kim[5] and Guangbin Ji[1,*]

[1] School of Materials Science and Technology, Nanjing University of Aeronautics and Astronautics, Nanjing, Jiangsu 210016, P. R. China

[2] School of Materials Science and Engineering, Nanjing University of Science and Technology, Nanjing, Jiangsu 210094, P. R. China

[3] School of Physics, Nanjing University of Science and Technology, Nanjing, Jiangsu 210094, P. R. China

[4] School of Mechanical Engineering, Nanjing University of Science and Technology, Nanjing, Jiangsu 210094, P. R. China

[5] School of Physics, Chungbuk National University, Cheongju 28644, South Korea

*Corresponding authors. E-mail: zhangyujing@njust.edu.cn (Yujing Zhang); ouyangboyi@njust.edu.cn (Bo Ouyang); gbji@nuaa.edu.cn (Guangbin Ji)

Supplementary Figures

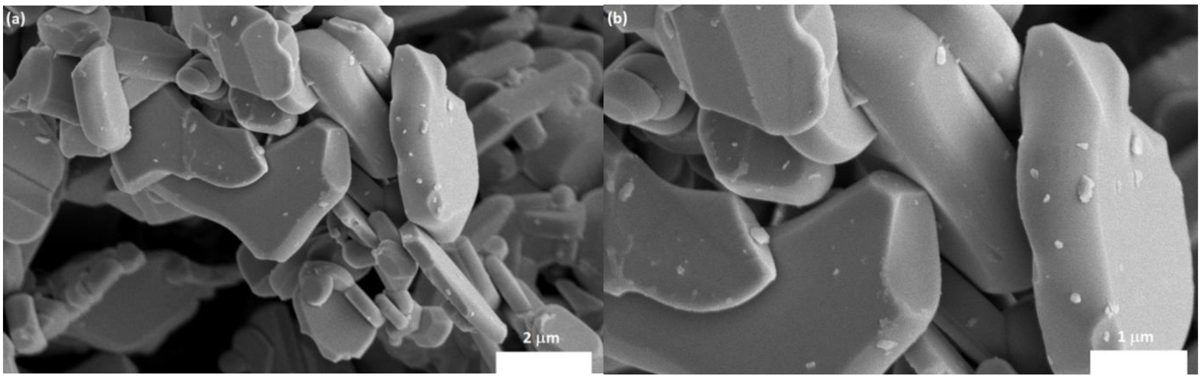

Fig. S1 SEM images of the original M-type barium ferrite at (**a**) 10,000x magnification and (**b**) 20,000x magnification

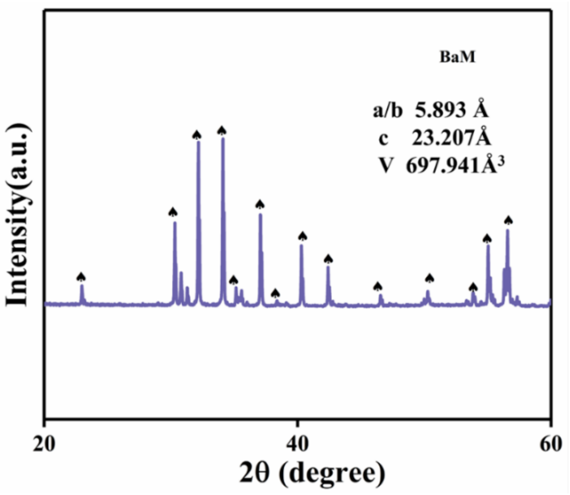

Fig. S2 XRD pattern and lattice parameters of the original M-type barium ferrite

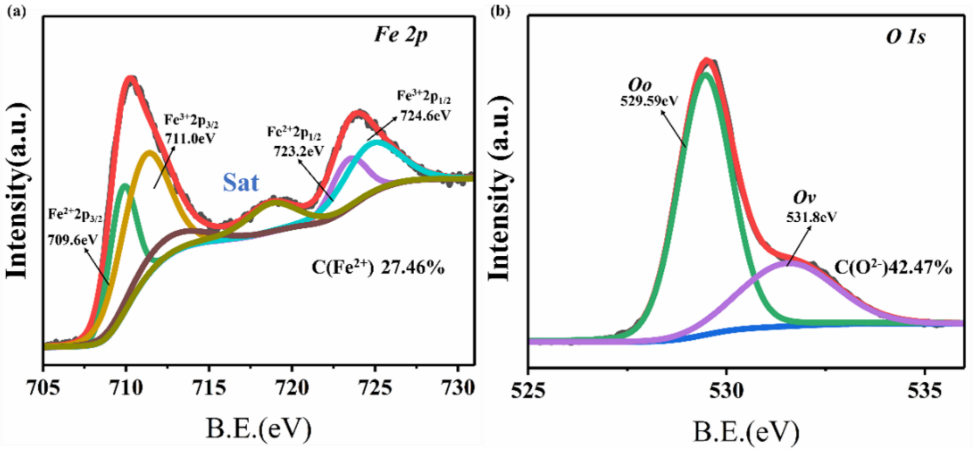

Fig. S3 XPS spectra for **(a)** Fe 2p and **(b)** O 1s of the original M-type barium ferrite

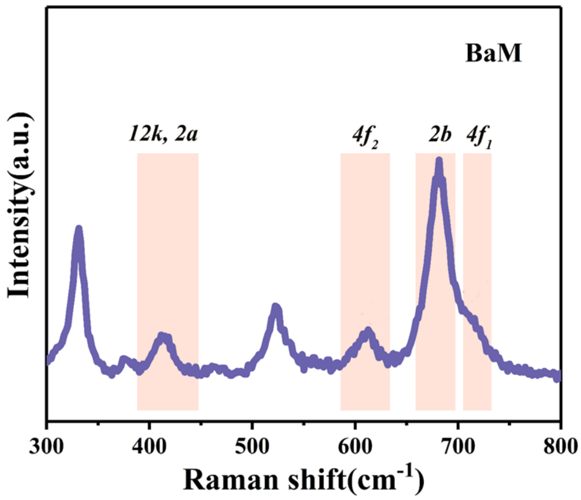

Fig. S4 Raman patterns of the original M-type barium ferrite

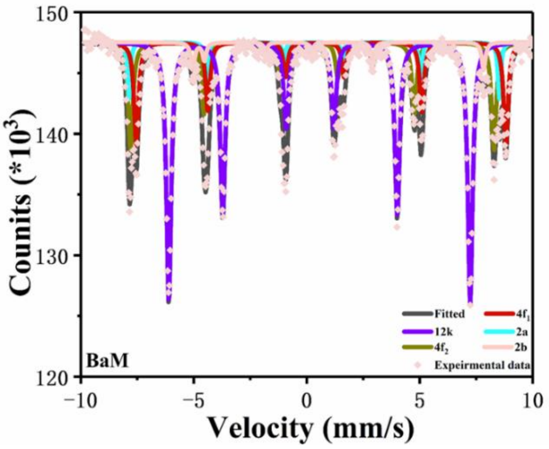

Fig. S5 Mössbauer spectra of the original M-type barium ferrite

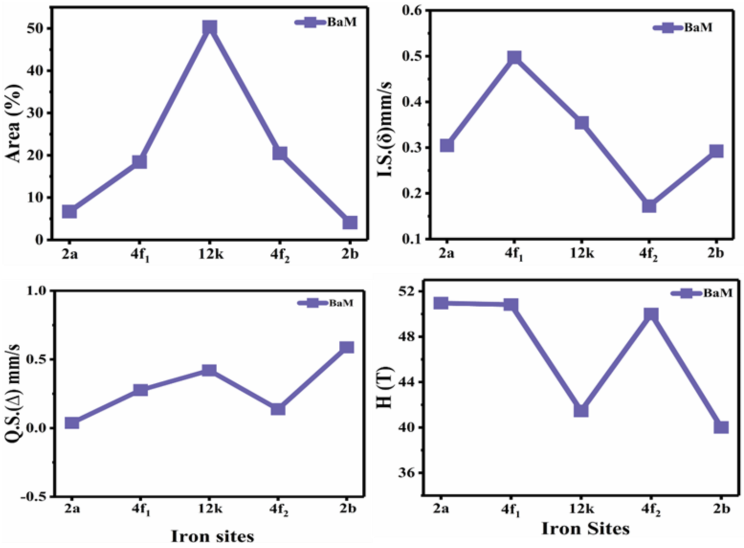

Fig. S6 Parameters of occupation area, I.S., Q.S., H_{hf} deduced from Mössbauer spectra of the original M-type barium ferrite

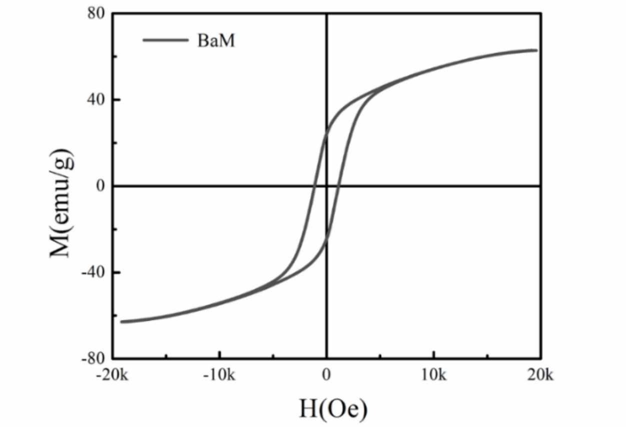

Fig. S7 Hysteresis loop of the original M-type barium ferrite

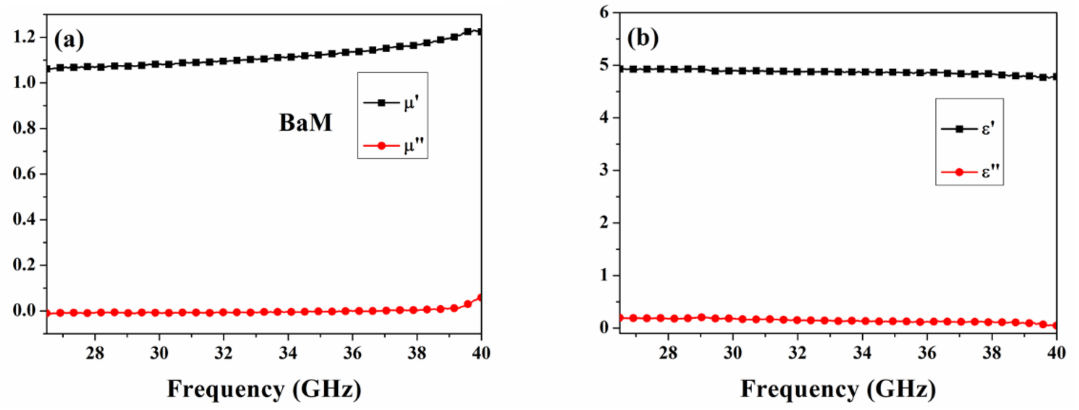

Fig. S8 Electromagnetic parameters of the original M-type barium ferrite

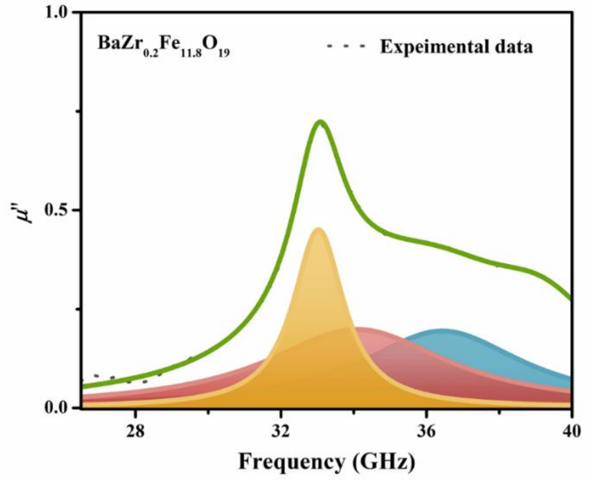

Fig. S9 Magnetic resonance peak-differentiating and imitating of the sample with composition of $BaZr_{0.2}Fe_{11.8}O_{19}$

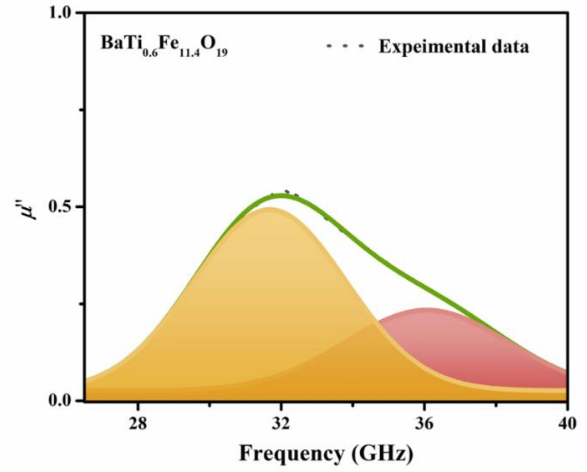

Fig. S10 Magnetic resonance peak-differentiating and imitating of the sample with composition of $BaTi_{0.6}Fe_{11.4}O_{19}$

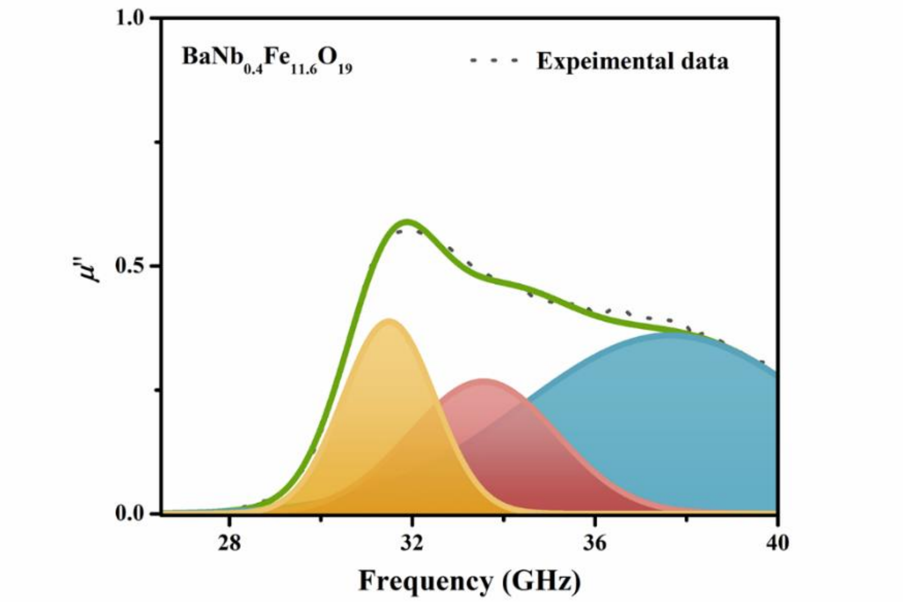

Fig. S11 Magnetic resonance peak-differentiating and imitating of the sample with composition of $BaNb_{0.4}Fe_{11.6}O$

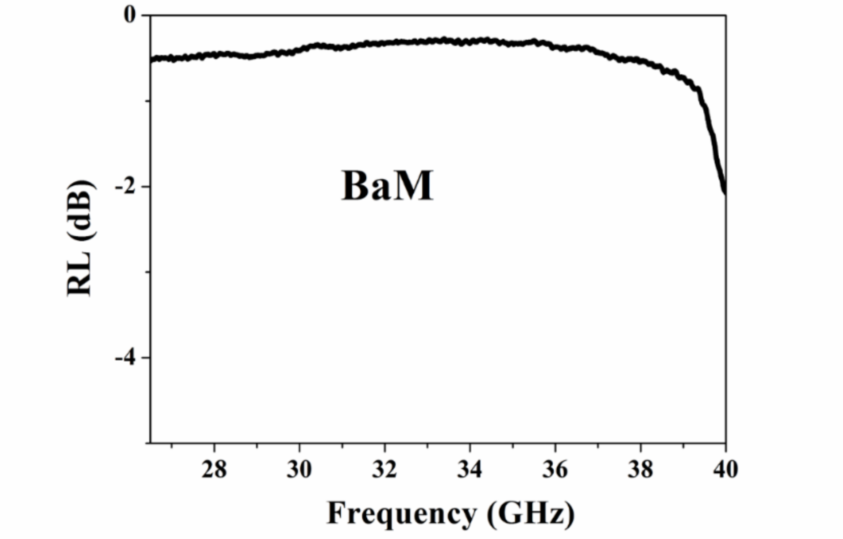

Fig. S12 Reflection loss of the original M-type barium ferrite

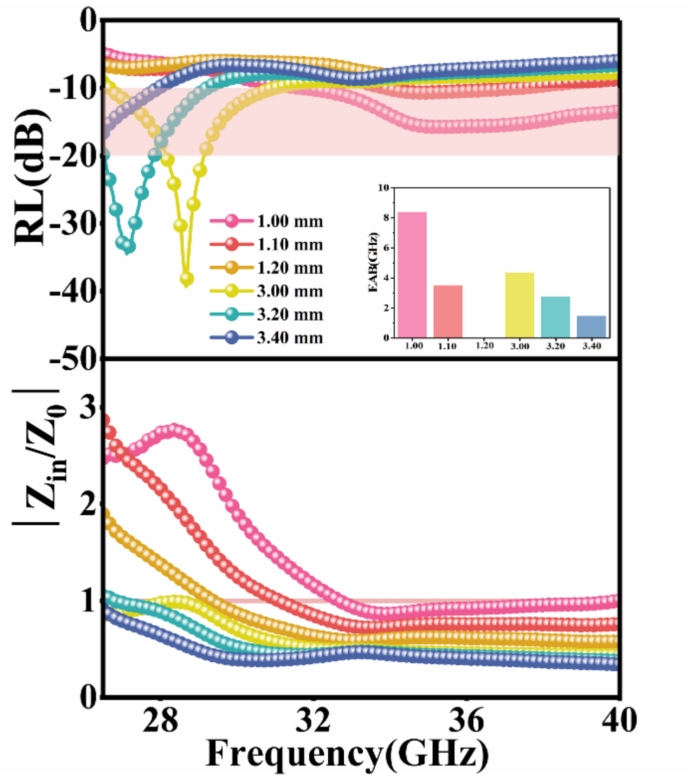

Fig. S13 2D reflection loss and impedance matching values of the $BaZr_{0.2}Fe_{11.8}O_{19}$ with thickness of 1.0 mm, 1.1 mm, 1.2 mm, 3.0 mm, 3.2 mm, 3.4 mm

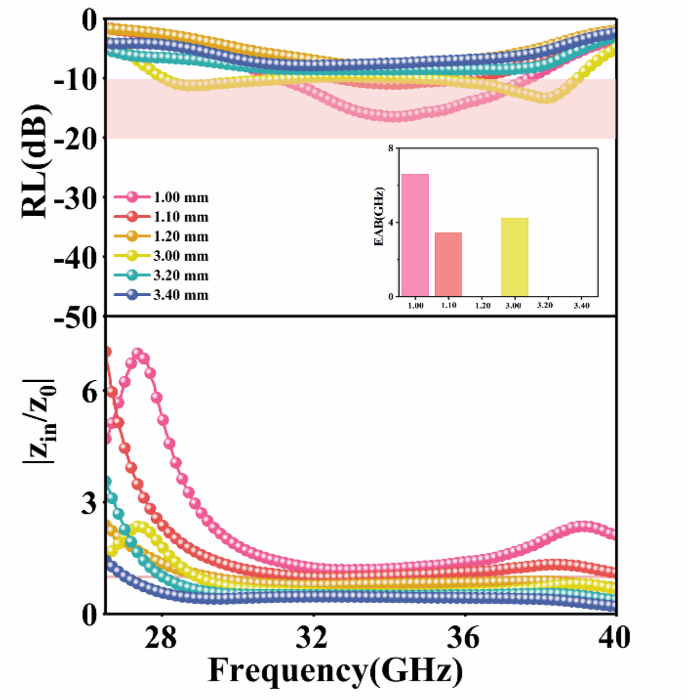

Fig. S14 2D reflection loss and impedance matching values of the $BaTi_{0.6}Fe_{11.4}O_{19}$ with thickness of 1.0 mm, 1.1 mm, 1.2 mm, 3.0 mm, 3.2 mm, 3.4 mm

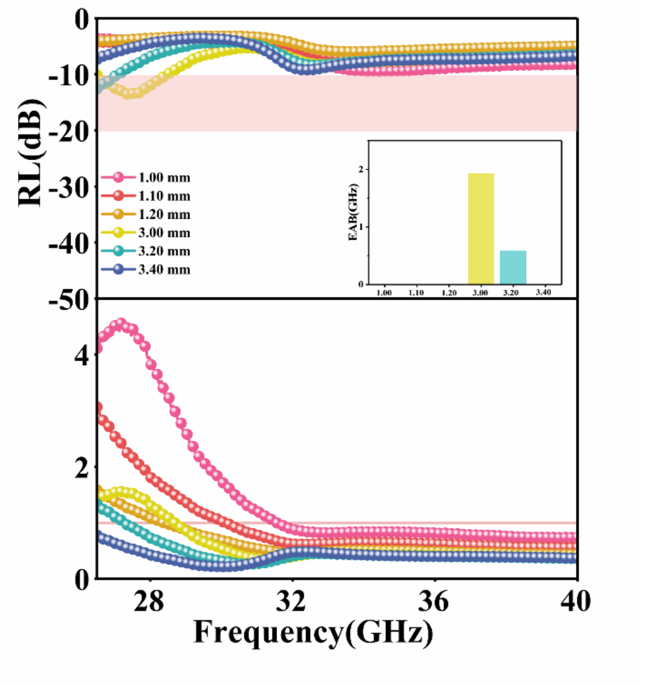

Fig. S15 2D reflection loss and impedance matching values of the $BaNb_{0.4}Fe_{11.6}O_{19}$ with thickness of 1.0 mm, 1.1 mm, 1.2 mm, 3.0 mm, 3.2 mm, 3.4 mm

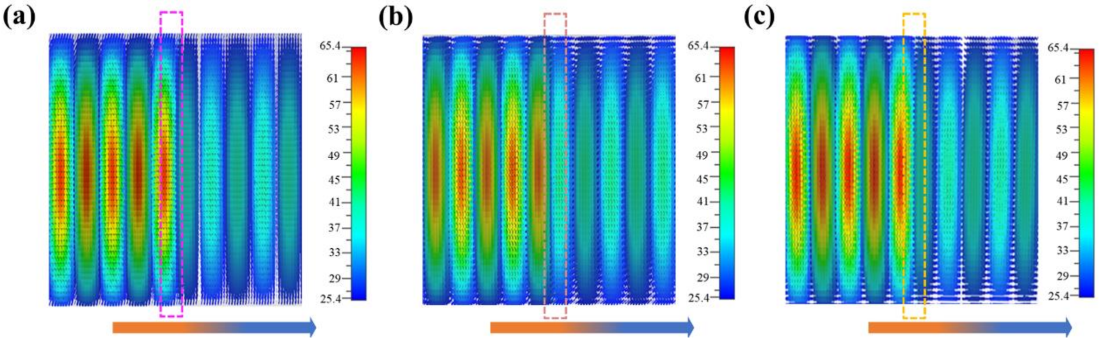

Fig. S16 Near-field simulations for (**a**) $BaZr_{0.2}Fe_{11.8}O_{19}$, (**b**) $BaTi_{0.6}Fe_{11.4}O_{19}$ and (**c**) $BaNb_{0.4}Fe_{11.6}O_{19}$

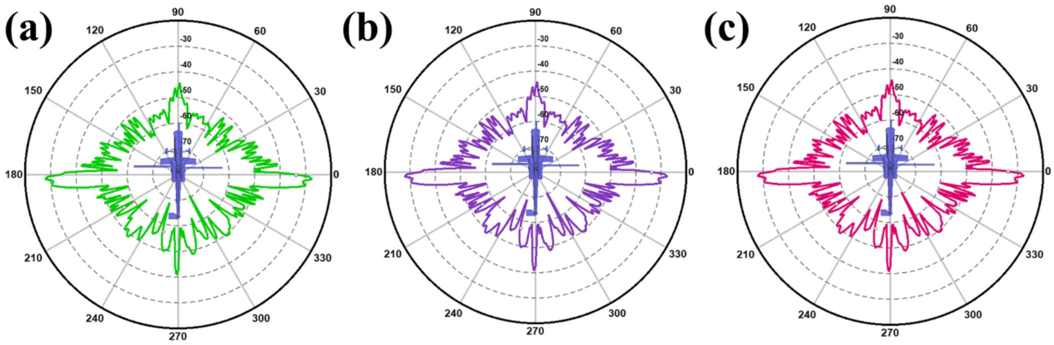

Fig. S17 Far-field simulations for bistatic scattering RCS Abs (**a**) $BaZr_{0.2}Fe_{11.8}O_{19}$, (**b**) $BaTi_{0.6}Fe_{11.4}O_{19}$ and (**c**) $BaNb_{0.4}Fe_{11.6}O_{19}$

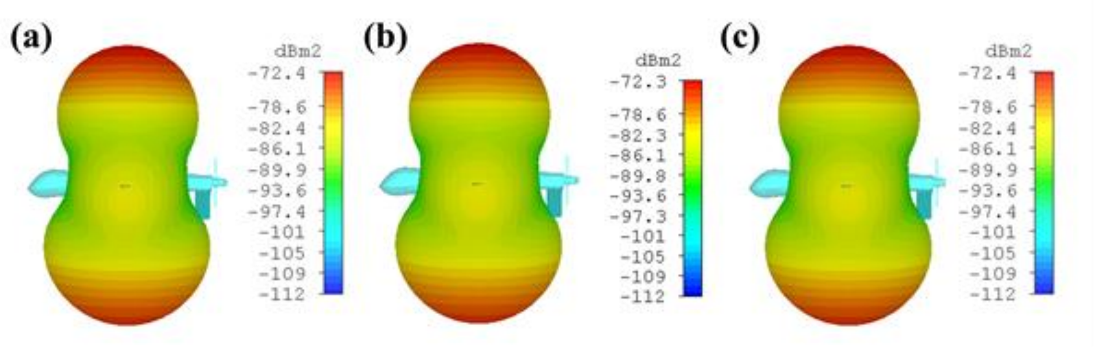

Fig. S18 Far-field simulations of 3D bistatic scattering RCS values for (**a**) $BaZr_{0.2}Fe_{11.8}O_{19}$, (**b**) $BaTi_{0.6}Fe_{11.4}O_{19}$ and (**c**) $BaNb_{0.4}Fe_{11.6}O_{19}$

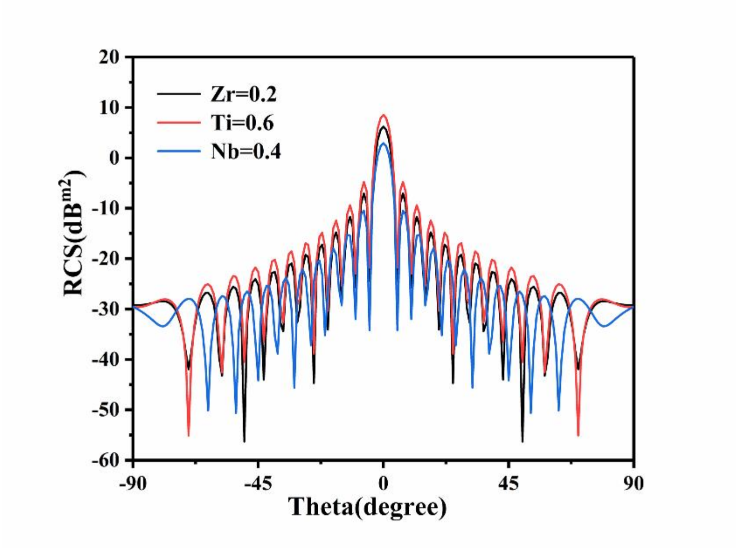

Fig. S19 Far-field simulations of 2D bistatic scattering RCS values for $BaZr_{0.2}Fe_{11.8}O_{19}$, $BaTi_{0.6}Fe_{11.4}O_{19}$ and $BaNb_{0.4}Fe_{11.6}O_{19}$
